# Daw1 regulates the timely onset of cilia motility during development

**DOI:** 10.1242/dev.200017

**Published:** 2022-06-16

**Authors:** Elizabeth A. Bearce, Zoe H. Irons, Samuel B. Craig, Colin J. Kuhns, Cynthia Sabazali, Dylan R. Farnsworth, Adam C. Miller, Daniel T. Grimes

**Affiliations:** 1 Institute of Molecular Biology, Department of Biology, University of Oregon, Eugene, OR 97403, USA; 2 Institute of Neuroscience, Department of Biology, University of Oregon, Eugene, OR 97403, USA

**Keywords:** Daw1, Motile cilia, Zebrafish, Axis development, Scoliosis, Primary ciliary dyskinesia

## Abstract

Motile cilia generate cell propulsion and extracellular fluid flows that are crucial for airway clearance, fertility and left-right patterning. Motility is powered by dynein arm complexes that are assembled in the cytoplasm then imported into the cilium. Studies in *Chlamydomonas reinhardtii* showed that ODA16 is a cofactor which promotes dynein arm import. Here, we demonstrate that the zebrafish homolog of ODA16, Daw1, facilitates the onset of robust cilia motility during development. Without Daw1, cilia showed markedly reduced motility during early development; however, motility subsequently increased to attain close to wild-type levels. Delayed motility onset led to differential effects on early and late cilia-dependent processes. Remarkably, abnormal body axis curves, which formed during the first day of development due to reduced cilia motility, self-corrected when motility later reached wild-type levels. Zebrafish larva therefore possess the ability to survey and correct body shape abnormalities. This work defines Daw1 as a factor which promotes the onset of timely cilia motility and can explain why human patients harboring *DAW1* mutations exhibit significant laterality perturbations but mild airway and fertility complications.

## Abstract (precis)

**Summary:** Daw1 promotes the onset of timely cilia motility for robust axial straightening during zebrafish development.

## INTRODUCTION

Motile cilia, microtubule-based organelles that protrude from the cell membrane, beat to drive cell propulsion and generate extracellular fluid flows. They are involved in several developmental and homeostatic processes (Spassky and Meunier, 2017) including airway clearance, left-right (L-R) organ asymmetry and fertility (Wallmeier et al., 2020). Zebrafish, *Danio rerio*, is a powerful model for elucidating roles of motile cilia in development and disease (Austin-Tse et al., 2013; Becker-Heck et al., 2011; Kramer-Zucker et al., 2005). Zebrafish mutants with perturbed motile cilia exhibit aberrant L-R patterning and do not generate cerebrospinal fluid (CSF) flow (Kramer-Zucker et al., 2005; Olstad et al., 2019; Thouvenin et al., 2020), resulting in failure to straighten the body axis over the first day of development and causing ventral axis curves referred to as ‘curly tail down’ (CTD) (Cantaut-Belarif et al., 2018; Zhang et al., 2018; Kramer-Zucker et al., 2005). Transiently restoring cilia motility to rescue CTD showed that motility is also required for the maintenance of spinal straightness during juvenile growth, with late disruption of motility causing three-dimensional spinal curves (Grimes et al., 2016). These phenotypes mimicked the prevalent human spine disorder idiopathic scoliosis (IS) in which curves manifest during adolescent growth (Bearce and Grimes, 2021; Boswell and Ciruna, 2017; Chen et al., 2016; Grimes et al., 2016).

These examples attest that motile cilia play diverse roles during development, growth and homeostasis, across varied time scales. During L-R patterning, ∼50-200 motile cilia act rapidly, over a few hours (Little and Norris, 2021). In other contexts, such as airway clearance, hundreds of thousands of cilia function, with a gradual turnover of ciliated cells, throughout life. As such, the requirements for timely initiation of motility after cilia emerge from cells is likely to be context-dependent. Yet, little is known about the regulation of when cilia begin to beat as, or after, they form. In *Chlamydomonas reinhardtii*, a unicellular algae, cilia achieve periodic beating when 4 µm long, approximately one third of their final length (Bottier et al., 2019). In the zebrafish Kupffer's vesicle (KV) (Essner et al., 2005; Kramer-Zucker et al., 2005), the majority of cilia are immotile at the three-somite stage but become motile by the eight-somite stage, 2.5 h later (Tavares et al., 2017). Similarly, airway cilia waveforms change as specific dynein arm motors are localized to the axoneme during cilia growth (Oltean et al., 2018). Discovering factors which regulate when cilia begin to beat robustly and determining their importance in cilia-dependent processes would lend support to a hypothesis that ‘beat onset’ is itself a regulatory step, beyond being some final consequence of cilia assembly.

Motile cilia consist of an axoneme of an outer ring of nine microtubule doublets and, usually, a central pair of microtubules (Ishikawa et al., 2021). Cilia form from basal bodies, modified centrioles, which have docked with the apical plasma membrane (Kumar and Reiter, 2021). They are built and maintained by intraflagellar transport (IFT), in which motors transport cargo along the axoneme (Kozminski et al., 1993; Lechtreck, 2015). Motility is generated by outer and inner dynein arms, which power cilia beat frequency and waveform, respectively (King, 2016; Oda et al., 2014). Outer dynein arms (ODAs) are assembled in the cytoplasm then imported into the cilium (Fowkes and Mitchell, 1998; Lechtreck, 2015; Omran et al., 2008). Import depends on ODA16, which physically bridges ODAs and the IFT-B complex protein IFT46 (Dai et al., 2018; Hou and Witman, 2017; Taschner et al., 2017). In *Chlamydomonas*, loss of ODA16 causes decreased axonemal localization of ODAs and defective motility (Ahmed and Mitchell, 2005; Ahmed et al., 2008). This function is conserved, with motile cilia-associated phenotypes being found in zebrafish, mice and planarian flatworms upon perturbation of ODA16 homologs (Gao et al., 2010; Lesko and Rouhana, 2020; Solomon et al., 2017). Some species-specific differences in ODA16 homolog structures also imply possible differences in the mechanics of ODA16 action (Wang et al., 2020). Moreover, ODA16 is not absolutely required for motility in some contexts; for example, in *Chlamydomonas*, ODA16 is not essential for ODA import, but instead enhances the efficiency of import (Ahmed and Mitchell, 2005; Ahmed et al., 2008).

While exploring the zebrafish ODA16 homolog, Daw1, we found that Daw1 controls the timely onset of cilia beating during development. Embryonic phenotypes were fully penetrant in *daw1* mutants but later phenotypes, including spinal curves, only occurred in a subset of mutants. Daw1-deficient zebrafish showed reduced cilia motility during the first day of development but, over the following days, motility increased to wild-type levels. Daw1 therefore ensures that timely and robust motility is achieved during development. The motility delay caused CTD during early stages which, remarkably, later self-corrected. Thus, zebrafish larvae can adapt to anatomical aberrations and re-organize to generate the ‘target morphology’ of a linear body. Overall, this demonstrates that Daw1 is a regulator of the timing of onset of cilia motility and establishes the requirements of this function in development.

## RESULTS AND DISCUSSION

**Fig. 1.**
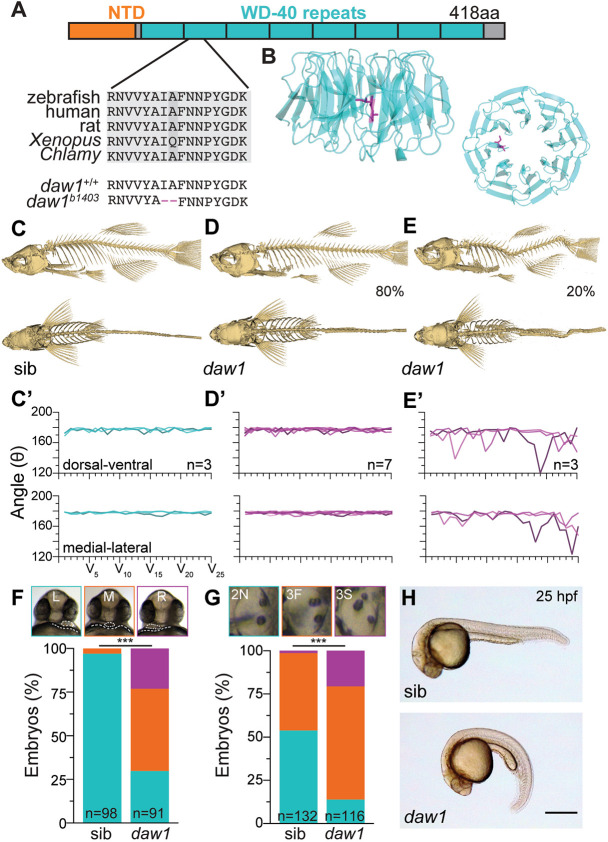
***daw1^b1403^* mutants exhibit motile cilia-associated defects.** (A) The 418 amino acid Daw1 protein with short N-terminal domain (NTD) and large β-propeller composed of WD40 repeats. *daw1^b1403^* deletes two conserved residues, I140 and A141. (B) Human DAW1 X-ray structure (Protein Data Bank: 5NNZ) showing I140 and A141 location. (C-E) 3D reconstitutions of µCT data. Most *daw1^b1403^* mutants showed normal spines (D), but 20% exhibited curves (E), compared with control (C). (C′-E′) Quantitation of curvature showed curved *daw1^b1403^* mutants had a non-stereotyped pattern (E′), compared with control (C′) and non-curved *daw1^b1403^* mutants. (F) Top: representative image of 25 hpf larvae showing the heart (dotted oval) on top of the yolk (dotted line). Bottom: *daw1^b1403^* mutants exhibited randomized heart laterality including left (L; green), middle (M; orange) and right (R; magenta)-facing hearts (*n*=91 mutants and 98 sib controls; ***P=4.84×10^−21^, chi square). (G) Top: otic vesicles at 25 hpf showed abnormal phenotypes in *daw1^b1403^* mutants [2N (green) – two normal; 3F (orange) – three otoliths, posterior two fused; 3S (magenta) – three separate otoliths. Bottom: quantification of *n*=116 mutants and 132 sibs; ***P=1.31×10^−12^, chi square. (H) CTD in *daw1^b1403^* mutants was fully penetrant at 25 hpf. Scale bar: 0.5 mm (H).

We generated a *daw1* mutant line harboring a deletion of two amino acids located centrally in a β-propeller structure (Fig. 1A,B; Fig. S1). This should disrupt Daw1 function as the β-propeller interacts with ODAs (Taschner et al., 2017). *daw1^b1403^* homozygous adults were viable and fertile; most showed no obvious phenotypes. However, ∼20% exhibited spinal curves in dorsal-ventral and medio-lateral directions (Fig. 1C-E′; Fig. S2). Curves occurred in the absence of abnormalities in vertebra shape or patterning, suggesting that they were not caused by structural vertebral defects (Figs S2,S3; Movie 1). Coupled with the 3D nature of the curves this suggested that phenotypic *daw1^b1403^* mutants exhibited characteristics of IS.

Although other cilia motility mutants are known to develop IS-like spinal curves (Grimes et al., 2016), *daw1^b1403^* mutants were unusual in that the penetrance of curves was low. We hypothesized this could be due to cilia motility being only partially affected in *daw1^b1403^* mutants. We first investigated this by determining the consequences of *daw1^b1403^* mutation in contexts beyond the spine, given that *daw1* is expressed in numerous motile ciliated tissues in the embryo (Fig. S4; Gao et al., 2010). *daw1^b1403^* mutants had high levels of L-R patterning defects and otolith placement abnormalities (Fig. 1F,G), two processes partly controlled by motile cilia (Colantonio et al., 2009; Grimes and Burdine, 2017). In addition, 100% of *daw1^b1403^* mutants exhibited CTD at 25 h postfertilization (hpf) (Fig. 1H), as also found in Daw1 morphants (Gao et al., 2010). Abnormal cilia motility-associated phenotypes were therefore fully penetrant in *daw1^b1403^* mutants during early development.

**Fig. 2.**
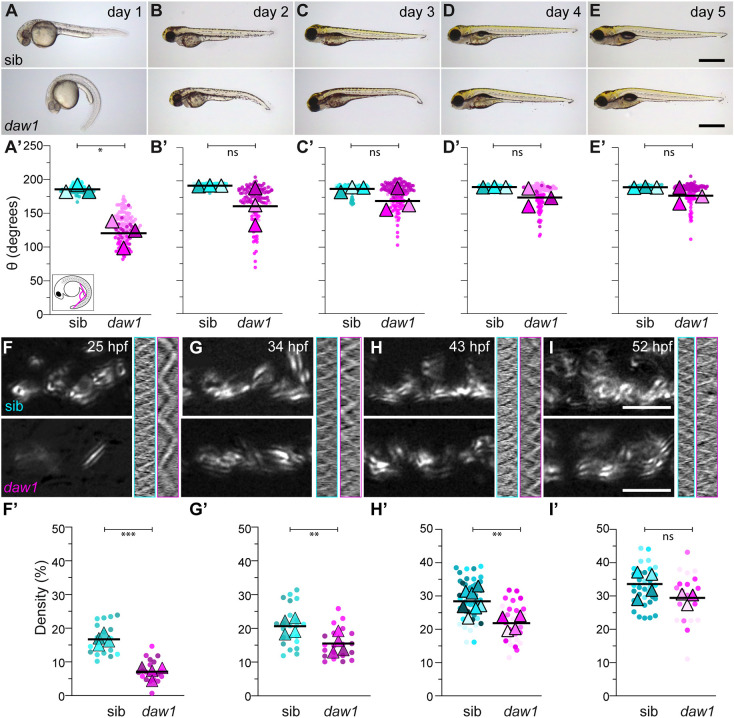
**Body axis curvature self-corrects after delayed onset cilia motility.** (A-E) The first 5 days of development show *daw1^b1403^* mutants self-correct curves. (A′-E′) SuperPlots of body angle, θ (inset in A′). Pairwise comparisons used two-way ANOVA adjusted for multiple comparisons. (F-I) TICS of CC cilia motility at different time points. (F′-I′) SuperPlots of the proportion of the CC covered by cilia motility (motile area). ***P<0.001, **P<0.01, *P<0.05 (two-tailed unpaired *t*-tests). ns, not significant. Scale bars: 0.5 mm (A-E); 5 µm (F-I).

To investigate why only 20% of *daw1^b1403^* mutants developed adult spinal curves, we followed mutants through development and growth. Mutants initially exhibited CTD, but most recovered to generate a linear axis by 3-5 days postfertilization (dpf) (Fig. 2A-E′), as shown by quantitation of body angle, θ. Although significantly different at 1 dpf, by 5 dpf, θ for mutants and controls was similar (177.8±12.1° for *daw1^b1403^* and 190.4±0.8° for controls, mean±s.d.; Fig. 2E′). Thus, the consequences of *daw1* mutation on body axis development are temporary, with mutants being able to self-correct early curves.

Although most recovered, some *daw1^b1403^* mutants retained axial kinks (Fig. 2E′; Fig. S5). To determine whether these caused later IS-like phenotypes, we followed kinked and straight mutants to adulthood. Of mutants that had attained straightness by 5 dpf, 0/28 (0%) showed spinal curves as adults (Fig. 1D,D′; Fig. S2B) but 23/65 (35%) mutants that exhibited kinks at 5 dpf developed curves (Fig. 1E,E′; Fig. S2C). Thus, spinal curves arose in mutants that failed to fully recover from CTD. This could explain why *daw1^b1403^* mutants exhibit a different, more variable, curve pattern compared with other cilia-associated IS genetic models in which spinal curves are caused by reduced cilia motility during juvenile growth (Grimes et al., 2016).

To understand how *daw1^b1403^* mutants self-corrected axial curves, we assessed motile cilia in the central canal (CC). Immunostaining showed normal length of CC cilia in mutants (Fig. S6), as expected (Gao et al., 2010). Although controls showed robust cilia beating (average frequency: 17.1±5.8 Hz) most cilia in *daw1^b1403^* mutants were static (Fig. 2F; Movie 2), something corroborated by quantitation of the area occupied by moving cilia, termed motile area (Fig. 2F′). The few cilia in mutants that showed motility beat slowly (7.4±2.7 Hz) and erratically (Fig. 2F; Fig. S7). Thus, the motility of CC cilia was significantly reduced in *daw1^b1403^* mutants at 25 hpf, explaining the fully penetrant CTD at this stage.

We hypothesized that self-correction of CTD might occur if CC cilia motility increased over time. Quantitation of motility at 34, 43, and 52 hpf indeed showed such an increase (Fig. 2G-I′; Fig. S7; Movies 3-5). By 52 hpf, motility was no longer statistically different in *daw1^b1403^* mutants compared with controls (Fig. 2I′), showing that close to wild-type levels of motility were ultimately achieved, with a delay of ∼1.5 days. Beyond the CC, motile cilia in the pronephric duct also displayed time-dependent phenotypes in *daw1^b1403^* mutants. At 25 hpf, mutant cilia beat more slowly and were disorganized compared with controls (Movies 6,7). By 34 hpf, motility was similar to controls and cilia were correctly bundled (Movies 8,9). By contrast, we found no differences in sperm flagellum motility from samples extracted from *daw1^b1403^* mutant males (Movies 10,11). This could be because short-term delays in motility onset are not evident in adult cilia. Last, cilia in KV were immotile in *daw1^b1403^* mutants (Movies 12,13), similar to what was previously shown in *daw1* morphants (Gao et al., 2010). This likely reflects that KV cilia are short-lived and have not had the necessary time to import ODAs in the absence of Daw1 function. Overall, this defines Daw1 as a factor which controls the timely onset of cilia motility on developmental timescales in zebrafish.

This idea coheres with the model that Daw1/ODA16 enhances the efficiency of ODA import in *Chlamydomonas* (Ahmed and Mitchell, 2005; Ahmed et al., 2008). We suggest that inefficient ODA import in *daw1^b1403^* mutants causes delayed onset of robust motility, explaining why motile cilia are more affected earlier in development (i.e. in KV as well as in the central canal and pronephros at earlier time points) than later. This can also explain why early motile cilia-dependent processes such as L-R patterning and initial axial straightening are severely impacted while later ones are not. Indeed, Daw1 morpholino knockdown caused significant motile cilia-associated defects in embryos (Gao et al., 2010); however, the transient nature of knockdown meant that the temporal requirements for Daw1 could not be assessed.

**Fig. 3.**
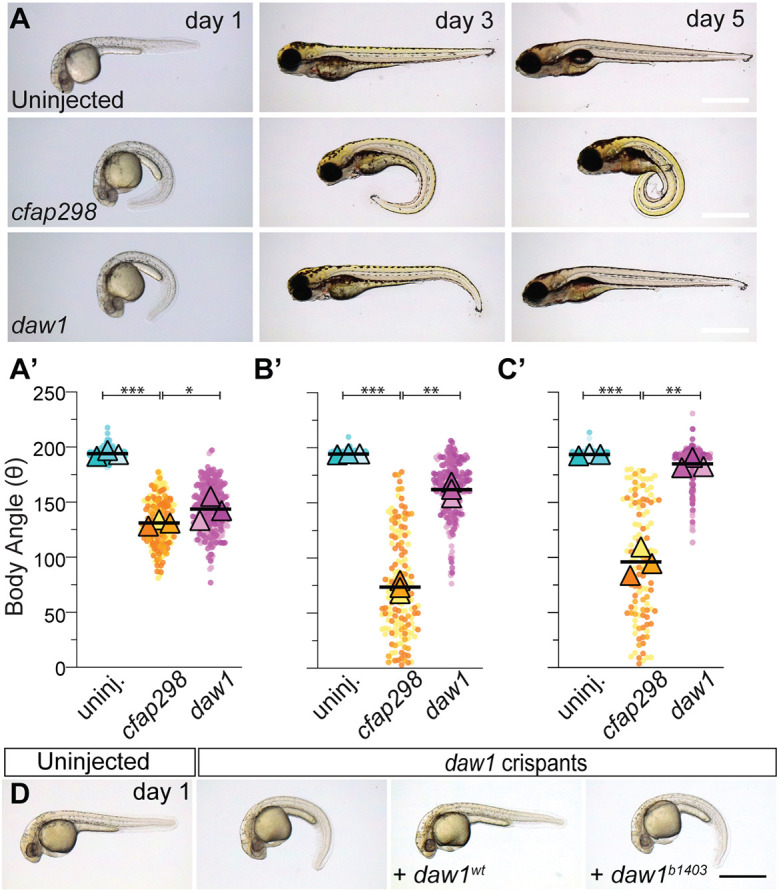
***daw1^b1403^* encodes a loss-of-function protein.** (A-C) Lateral views of controls and *daw1* and *cfap298* crispants. (A′-C′) SuperPlots of body angle quantitation. Pairwise comparisons used two-way ANOVA adjusted for multiple comparisons. ***P<0.001, **P<0.01, *P<0.05. ns, not significant. (D) Expression of Daw1^WT^ but not Daw1^b1403^ rescues the body curves of *daw1* crispants. Scale bars: 0.5 mm (A-D).

It is also possible that Daw1 is essential for the levels of ODA import needed for robust motility at all stages, but that the *daw1^b1403^* line encodes a hypomorphic mutation that reduces but does not abolish Daw1 function. As *daw1^b1403^* encodes a two amino acid deletion, it is conceivable that mutant protein retains some function. Both models could account for delayed motility in *daw1^b1403^* mutants. To distinguish, we targeted *daw1* with multiple guide RNAs (gRNAs), generating crispant embryos with mosaic mutations. *daw1* crispants showed CTD at 1 dpf (Fig. 3A,A′; Fig. S8A,B) then self-corrected between days 2 and 5. The majority (91.2±6.1%) achieved a linear body axis by 5 dpf (Fig. 3B-C′). Using the same approach to target *cfap298*, an ODA assembly factor required for motility (Austin-Tse et al., 2013; Jaffe et al., 2016), led to *cfap298* crispants which both developed and retained CTD (Fig. 3A-C′). In addition, expression of mRNA coding for wild-type Daw1 rescued CTD in *daw1* crispants, demonstrating the specificity of the phenotype induced by *daw1* gRNAs (Fig. 3D; Fig. S8C). By contrast, expression of the Daw1 *b1403* variant did not rescue crispants (Fig. 3D; Fig. S8D). Last, we generated an additional mutant line, called *daw1^b1422^*, in which a large deletion and premature truncation codon occur (Fig. S9A-C). *daw1^b1422^* mutants also exhibited CTD, which self-corrected over time (Fig. S9D-D′). Together, this suggests that *daw1^b1403^* indeed represents a strong loss of function. We therefore favor the model that, by enhancing the rate of ODA import, Daw1 controls the timely onset of robust cilia motility during development.

What might be the implications for human disease? The major motile ciliopathy is primary ciliary dyskinesia (PCD) which afflicts 1 in 10,000 people and is characterized by situs abnormalities, reduced mucus clearance leading to airway infections and infertility (Wallmeier et al., 2020). Our model for Daw1 function, in which embryonic cilia are significantly disrupted but cilia at later stages are not, suggests that putative *DAW1* patients would exhibit minor respiratory or fertility clinical manifestations but severe organ situs complications. This is because ciliated epithelial cells of the respiratory and ovarian tracts comprise, together, hundreds of thousands of cilia which are relatively long-lived, with ciliated cells exhibiting gradual turnover. A potential short-term delay in motility due to slower ODA import after new cilia form within a single region of these ciliated tracts is therefore unlikely to have a substantial impact on organ function nor lead to severe disease. By contrast, the motile cilia in the embryonic node that are crucial for L-R patterning form, function and then regress on the timescale of hours (Little and Norris, 2021), and so rapid onset of motility is essential. In agreement, a *Daw1* mouse mutant exhibited severe L-R patterning defects but relatively minor lung cilia motility abnormalities (Solomon et al., 2017), although mucociliary clearance was still defective in these mutants, suggestive of reduced quality of cilia beating. Moreover, in an accompanying manuscript, we report human patients harboring loss-of-function *DAW1* mutations in which situs defects were prominent but respiratory and fertility complications were absent or mild (Leslie, J. S., Hjeij, R., Vivante, A., E. A. B., Dyer, L., Wang, J., Rawlins, L., Kennedy, J., Ubeyratna, N., Fasham et al., unpublished).

**Fig. 4.**
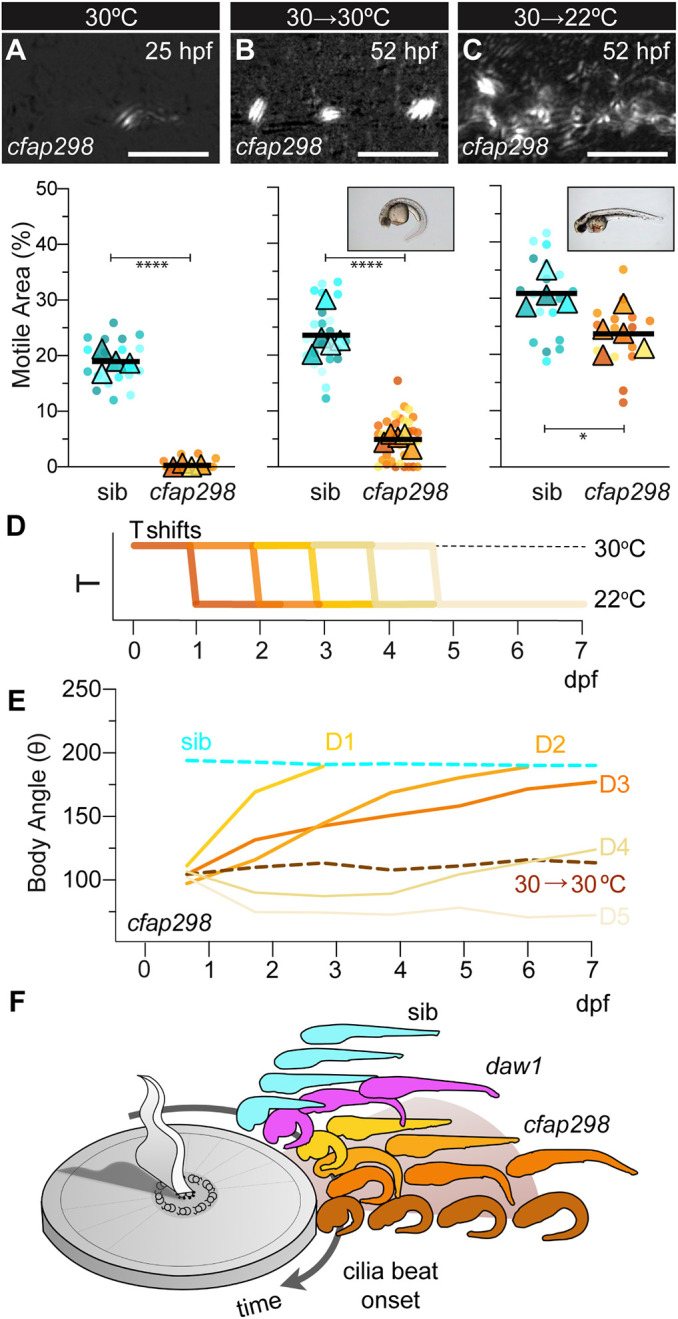
**Self-correction of curves can occur throughout developmental stages.** (A-C) Top: TICS of CC cilia motility of 25 hpf *cfap298^tm304^* mutants raised at 30°C (A), then 52 hpf after maintaining 30°C (B) or downshifting to 22°C at 24 hpf (C). Bottom: SuperPlots of motility data. Insets: axis straightening occurred in downshifted embryos but not those maintained at 30°C. (D) Schematic of temperature (T) shifts. Embryos were raised at 30°C until either 1, 2, 3, 4 or 5 dpf, then downshifted to 22°C, inducing cilia motility. Body angles were assessed until 7 dpf. (E) Body angles across 1-7 dpf after temperature downshifts on days 1-5 (D1-D5). (F) Summary showing zebrafish larvae self-correct curves after robust CC cilia motility onsets. Distance around the sundial represents later times of motility onset in distinct conditions. ****P<0.0001, *P<0.05 (two-tailed unpaired *t*-tests). Scale bars: 5 µm (A-C).

Last, the self-correction of CTD in *daw1* loss of function demonstrated the remarkable ability of zebrafish larvae to remodel their shape to achieve the species typical anatomy. To test whether this self-correction was caused by the late onset of cilia motility, we used a temperature-sensitive *cfap298^tm304^* mutant line (Jaffe et al., 2016). At restrictive temperatures, *cfap298^tm304^* mutants exhibited reduced CC cilia motility (Fig. 4A; Movie 14) and ventral curves (Fig. S10A,A′); at permissive temperatures, cilia were motile and embryos underwent straightening (Fig. S10; Movie 14; Jaffe et al., 2016). We performed restrictive-to-permissive temperature shifts to initially inhibit cilia motility but then later activate it (Fig. 4D). Downshifting at 24 hpf led to robust cilia beating by 52 hpf (Fig. 4B,C; Movies 15,16); the majority of larvae corrected CTD (Fig. 4B,C,E; Fig. S11). By contrast, larvae maintained at restrictive temperatures showed no recovery of motility and retained CTD (Fig. 4B,E; Movie 16). As a side note, a small proportion of mutants straightened at restrictive temperatures. This was partly dependent on whether or not mutants inherited wild-type *cfap298* gene product from their mothers (Fig. S10B). Overall, in both *daw1^b1403^* and downshifted *cfap298^tm304^* mutants, larvae responded by remodeling their abnormal shape to achieve the wild-type anatomy despite missing the developmental window during which axial straightening normally occurs.

To determine whether the ability to self-correct CTD was maintained as larvae aged, we performed temperature shifts at progressive times (Fig. 4D). Downshifting at 1 dpf resulted in axial straightening in 89.5±18.3% of larvae by 24 h post-shift and 99.1±1.6% by 48 h post-shift. Downshifting at 2 and 3 dpf led to progressively less efficient straightening (Fig. 4D; Fig. S11), though the majority of mutants (80.0±17.8%) downshifted even at 3 dpf still attained axial straightness by 4 days post-shift. Downshifting at 4 and 5 dpf led to inefficient straightening, with larvae largely being unable to attain a linear axis by the termination of the experiment at 7 dpf (Fig. 4D; Fig. S11).

This demonstrates the ability of early zebrafish larvae to remodel their shape to a ‘target morphology’ despite diverse starting conditions. During 1-3 dpf, larvae are not normally curved, yet, once cilia motility initiates, curved larvae resolve anatomical abnormalities to generate the wild-type form (Fig. 4F). This underscores the importance of viewing development as a flexible process that can respond to perturbations, rather than some programmed unfolding of genetically predetermined events. ‘Goal state’ models in which the organism is viewed as trying to achieve an anatomical set point, can be useful for understanding such phenotypes (Levin, 2021). Zebrafish axial straightening represents a tractable system to delve deeper into the multi-scale processes which sense the current anatomical state and implement changes that move the state towards the target set point.

Overall, we find that Daw1 is not essential for cilia motility per se, but controls the timely onset of motility in zebrafish. Early embryonic functions for motile cilia are highly dependent on Daw1, but later functions are not. These findings explain the severe laterality defects but mild lung and fertility complications in patients harboring *DAW1* mutations.

## MATERIALS AND METHODS

### Zebrafish

Zebrafish (*Danio rerio*) of the AB strain were used. Embryos from natural matings were incubated at 28°C unless otherwise stated. Zebrafish mutant lines used were *cfap298^tm304^* (Jaffe et al., 2016) and *daw1^b1403^* (this manuscript). Experiments were undertaken in accordance with research guidelines of the International Association for Assessment and Accreditation of Laboratory Animal Care and approved by the University of Oregon Institutional Animal Care and Use Committee.

### *daw1^b1403^* generation

Cas9 mRNA was generated by *in vitro* RNA synthesis using pCS2-nCas9n (Addgene #47929; Jao et al., 2013) as a template. For *daw1^b1403^*, a gRNA oligo of sequence 5′-TAATACGACTCACTATAGGAAGGGTTGTTGAAGGCGAGTTTTAGAGCTAGAA-3′ (Integrated DNA Technologies) targeting exon 5 of *daw1* was designed using CRISPRscan (Moreno-Mateos et al., 2015). Full gRNA templates were assembled by annealing and extension with a bottom strand oligo of sequence 5′-AAAAGCACCGACTCGGTGCCACTTTTTCAAGTTGATAACGGACTAGCCTTATTTTAACTTGCTATTTCTAGCTCTAAAAC-3′ using Taq Polymerase (New England Biolabs, M0273) and cycling parameters of 95°C (3 min), 95°C (30 s), 45°C (30 s), 72°C (30 s), 72°C (10 min) with 30 cycles of the central three steps. gRNA templates were purified with a DNA Clean & Concentrator Kit (Zymo Research, D4013) then subjected to *in vitro* RNA synthesis using a MEGAshortscript T7 Transcription Kit (Thermo Fisher Scientific, AM1354). Synthesized gRNAs were treated with 2 U of TURBO DNase (Thermo Fisher Scientific, AM2238) for 15 min at 37°C, purified using an RNA Clean & Concentrator Kit (Zymo Research, R1013) then aliquoted and stored at −80°C. For mutagenesis, 150 pg of gRNA and 100 pg of Cas9 mRNA were injected into the yolk at the one-cell stage. Injected embryos were screened for indels at the target site by PCR of extracted DNA with oligos 5′-CCTTCACTTTCCGTCTGTTTGCAG-3′ and 5′-GGGACAAGTGCTCCTGTTATGACTC-3′ followed by restriction digestion with HpyAV (New England Biolabs, R0621). Injected embryos were raised and outcrossed to establish F1 families from which individuals were outcrossed to generate F2 families with defined mutations. The *daw1^b1403^* six base pair deletion was identified by Sanger sequencing (GeneWiz).

### CRISPR/Cas9 mosaic mutagenesis

The four gRNA oligos used for *daw1* and *cfap298*, which contained gRNA target sites and adapter sequences, were chosen from a look-up table (Wu et al., 2018): *daw1* oligo sequences were: 5′-TAATACGACTCACTATAGGTCACCTGCTCGACACAGGGTTTTAGAGCTAGAAATAGC-3′; 5′-TAATACGACTCACTATAGGGCGGTGCTGTTACCCGTAGTTTTAGAGCTAGAAATAGC-3′; 5′-TAATACGACTCACTATAGGGTCTATTATAGGTATGCCGTTTTAGAGCTAGAAATAGC-3′ and 5′-TAATACGACTCACTATAGGCTGCTTGCGTATAGGTGTGTTTTAGAGCTAGAAATAGC-3′; and *cfap298* oligo sequences were 5′-TAATACGACTCACTATAGGTTCTCTTCAACACTACGGGTTTTAGAGCTAGAAATAGC-3′; 5′-TAATACGACTCACTATAGGGCTCCACAATCTGATCATGTTTTAGAGCTAGAAATAGC-3′; 5′-TAATACGACTCACTATAGGCATTCTTATTGGATCATGGTTTTAGAGCTAGAAATAGC-3′ and 5′-TAATACGACTCACTATAGGTCTCTGGCAGGTGCGCCCGTTTTAGAGCTAGAAATAGC-3′. gRNAs were synthesized from oligos in multiplex. First, oligos were pooled (10 μM) then annealed and extended with 10 μM of a bottom strand ultramer of sequence 5′-AAAAGCACCGACTCGGTGCCACTTTTTCAAGTTGATAACGGACTAGCCTTATTTTAACTTGCTATTTCTAGCTCTAAAAC-3′ using Phusion HF PCR Mastermix (New England Biolabs, M0531) and Phusion High-Fidelity DNA Polymerase (New England Biolabs, M0530) in a 50 μl reaction using cycling parameters of 98°C (2 min), 50°C (10 min), 72°C (10 min). Assembled oligos were purified using a DNA Clean & Concentrator kit (Zymo Research, D4013) then subjected to *in vitro* RNA synthesis using a HiScribe T7 High Yield RNA Synthesis kit (New England Biolabs, E2040). Synthesized RNA solution was treated with 2 U of TURBO DNase (Thermo Fisher Scientific, AM2238) for 15 min at 37°C then purified using an RNA Clean & Concentrator kit (Zymo Research, R1013). Synthesized gRNAs were aliquoted and stored at −80°C. For mutagenesis, 1000 pg of gRNA mix alongside 1600 pg/nl Cas9 (Integrated DNA Technologies, 1081058) were injected into the yolk of embryos at the one-cell stage of development.

### *daw1^b1422^* generation

*daw1^b1422^* mutants were isolated from clutches injected with four gRNAs (as in the ‘CRISPR/Cas9 mosaic mutagenesis’ section). Mutants were analyzed after outcrossing to at least the F3 generation. All four CRISPR sites were sequenced to identify mutations. The mutation occurred between gRNAs 2 and 3, with the 1 and 4 gRNAs not inducing mutations in this line. *daw1^b1422^* mutants were genotyped by PCR using primers 5′-CTCCCCTTCACTTTCCGTCT-3′ and 5′-GGTGTCCATGCTGCCTGTA-3′ leading to a 735 bp band in wild type and an ∼400 bp band in mutants.

### Larval body curve quantitation

Lateral views of zebrafish larvae were captured using a Leica S9i stereomicroscope with an integrated 10-megapixel camera. Body angles, θ, were measured in ImageJ (Schindelin et al., 2012) after double-blinding. For statistical analysis of changes in θ over time, we used the repeated measures two-way analysis of variance (ANOVA) adjusted for multiple comparisons by the two-stage linear step-up procedure of Benhamini, Krieger and Yekutieli.

### Calcein staining

Larvae were transferred to water containing 0.2% calcein (Sigma-Aldrich, C0875) for 10 min then briefly rinsed in water 2-3 times for 5 min each. Larvae were then immobilized with 0.005% tricaine, mounted in 0.8% low melt agarose with tricaine dropped on the surface and imaged with a Leica Thunder stereoscope.

### Immunofluorescence

Embryos and larvae were euthanized and fixed in 4% paraformaldehyde at 4°C for at least 16 h. If necessary, embryos were manually dechorionated before fixation. Samples were rinsed three times in 100% methanol and then placed in 100% methanol for 2 h before being rehydrated with 5 min rinses in 75% methanol in PBST (1× PBS with 0.1% Tween-20), 50% methanol in PBST, 25% methanol in PBST and PBST. After three more 5 min washes in PBST, samples were blocked in 5% normal sheep serum (NSS) and 1% DMSO in PBST for 2 h at room temperature. Samples were then incubated in primary antibodies anti-γ-tubulin (rabbit polyclonal, 1:600, Sigma-Aldrich, T5192) and anti-acetylated α-tubulin (mouse monoclonal, 1:500, Sigma-Aldrich, T6793) in 1% NSS and 1% DMSO in PBST at 4°C for 16 h, then washed five times for 30 min per wash in 1% NSS, 1% DMSO and 0.1 M NaCl in PBST. Samples were incubated in secondary antibodies goat anti-rabbit conjugated with Alexa Fluor 546 (1:500, Thermo Fisher Scientific, A-11035) and goat anti-mouse conjugated with Alexa Fluor 488 (1:500, Thermo Fisher Scientific, A-11001) and Hoechst (1:1000, Sigma-Aldrich, 94403) at 4°C for 16 h in the dark. Samples were washed five times for 30 min per wash in 1% NSS, 1% DMSO and 0.1 M NaCl in PBST, then dissected to remove head and yolk. Trimmed samples were slide-mounted in a lateral orientation in Aqua-mount mounting medium (Polysciences, 18606) under #1.5 coverslips. Laser scanning confocal imaging was performed on a Zeiss 880 inverted microscope with a 63×, 1.4 NA oil-immersion objective and PMT and GaAsP-PMT detectors. Pinholes were set to 1 airy unit with gain between 200-300 (GaAsP-PMT) and 550-700 (PMT); *x/y* pixel size was 0.0851964 μm. Images were processed and quantified using ImageJ. Cilia lengths were manually traced using the Freehand Line tool (*n*=50 per image).

### Live imaging of cilia

For central canal and pronephros imaging, 25-52 hpf embryos were manually dechorionated and anesthetized with tricaine. Embryos were laterally mounted in #1.5 coverslip-bottomed Mattek chambers by embedding in tricaine-laced, low-melt 0.5% agarose made in embryo medium. A Nikon Ti2 inverted microscope equipped with a Plan Apo VC 60× WI DIC 1.2 NA objective and pco.edge sCMOS camera was used to capture 512×512 DIC images in time series, at 250 frames per second for 4 s (1000 frames total). Pixel size was 0.11 μm. Images were processed and quantified using ImageJ. Time course data was rotated and cropped to isolate the CC. A moving average of 55 frames was subtracted from each frame (Stowers Institute ImageJ Plugins>jay_unruh>Detrend>Subtract Moving Average), and a Gaussian blur of 0.8 was applied to all frames. For manual analysis, line profiles were drawn through motile cilia, and kymographs were generated using KymographBuilder. For Fourier Transform-based analysis, image stacks were converted to 16-bit and processed with a Temporal Image Correlation Spectroscopy (TICS) package (Stowers>jay_unruh>ICS>stack fft TICS jru v1, analysis length=512). The first frame of each TICS series was deleted to account for sCMOS camera noise, and frequencies were measured from regions that overlapped cilia using the multi-measure *z*-axis profile tool (BAR>Analysis>Time Series>Multi ROI Profiler).

### Sperm extraction and imaging

Sperm were extracted from wild-type or homozygous mutant *daw1^b1403^* males, as described previously (Yamaguchi et al., 2018). Sperm were stored in 1/5 Hank's buffer at 4°C. A drop was applied to a 35 mm imaging chamber with #1.5 untreated coverslips, uncoated to encourage sperm head adherence. A perfusion chamber was created with a second coverslip and vacuum grease, and embryo medium was flushed through to activate sperm motility. Imaging was performed at 1 ms intervals for 750 ms, using a Nikon Ti2 inverted microscope as above.

### Constructs and mRNA overexpression

Daw1 cDNA was cloned from a cDNA library generated from RNA collected at 1 dpf using a Direct-Zol RNA kit (Zymo, R2051). mCherry-Daw1 was shuttled into pCS-DEST. Site directed mutagenesis of mCherry-Daw1 constructs was performed with a Q5 Site-Directed Mutagenesis kit (New England Biolabs, E0554S), using primers 5′-TTCAACAACCCTTACGGAG-3′ and 5′-GGCATACACAACGTTCCTG-3′. mCherry-Daw1 mRNA was synthesized from plasmid DNA after restriction enzyme digestion with SacII (New England Biolabs, R0175S). Linearized plasmids were purified using a DNA Clean & Concentrator kit (Zymo Research, D4013) then used as a template for *in vitro* mRNA synthesis using an mMessage mMachine SP6 Transcription kit (Thermo Fisher Scientific, AM1340). mRNA was purified using lithium chloride and stored at −80°C. For overexpression, 20 pg of mRNA was injected into the blastocyst at the one-cell stage of development.

### Single cell RNA-sequencing analysis and visualization

Single cell analysis was performed using a Zebrafish Single Cell Transcriptome Atlas covering 1, 2 and 5 dpf time points (Farnsworth et al., 2020). Sequencing data were analyzed using the Cell Ranger pipeline (Zheng et al., 2017), and Seurat software (Satija et al., 2015), with reads aligned to the zebrafish genome, GRCz11_93. UMAP analysis produced 220 clusters from 44,102 cells. Differential gene expression analysis used the FindAllMarkers function in Surat and the Wilcoxon rank sum test.

### X-ray microcomputed tomography

X-ray microcomputed tomography (µCT) scans were performed using a vivaCT80 (Scanco Medical) at an 18.5 µm voxel resolution. Single volume surface reconstitutions and DICOM files of individual fish were generated using Scanco software, imported into ImageJ and resliced (Images>Stacks>Reslice[/]) into lateral and dorsal views. Maximum intensity projections were generated for quantitation of spinal curves.

### Quantitation of spinal curves

To quantify spinal curves, *x*, *y* coordinate points were defined at the center of mass of each vertebra in either lateral or dorsal max projections. Additional ‘origin’ and ‘terminal’ coordinates were placed on the center of the Weberian vertebrae (rostral to vertebra 1) and the caudal fin (caudal to vertebra 25), so that angles could be measured for all vertebrae in the spinal column. The distance formula was used to calculate distance between consecutive vertebrae, and the law of cosines was applied to determine angular displacement of a single vertebra relative to previous and subsequent vertebrae. In straight segments, where these inputs constructed an impossible triangle, consecutive points with less than 36 µm (2 pixels) of deviation in the direction perpendicular to the long body axis were assigned 180° angles by default.

### Plotting

All plots, including SuperPlots (Lord et al., 2020), were generated using GraphPad version 8.4.3, except UMAP plots, which were generated in R. For plots showing body angles, dots represent individual embryos; triangles represent clutch averages and black lines show population means. For plots showing motile area, dots represent distinct regions of interest within an individual, triangles represent averages of data from different individuals and black lines show means.

## Supplementary Material

Supplementary information

Reviewer comments
