# Supplementary material for: Daw1 regulates the timely onset of cilia motility during development
Source: Development. 2022 Jun 16;149(12):dev200017. doi: 10.1242/dev.200017 (PMC9270974; doi:10.1242/dev.200017)
Supplement: Supplementary information [file develop-149-200017-s1.pdf]

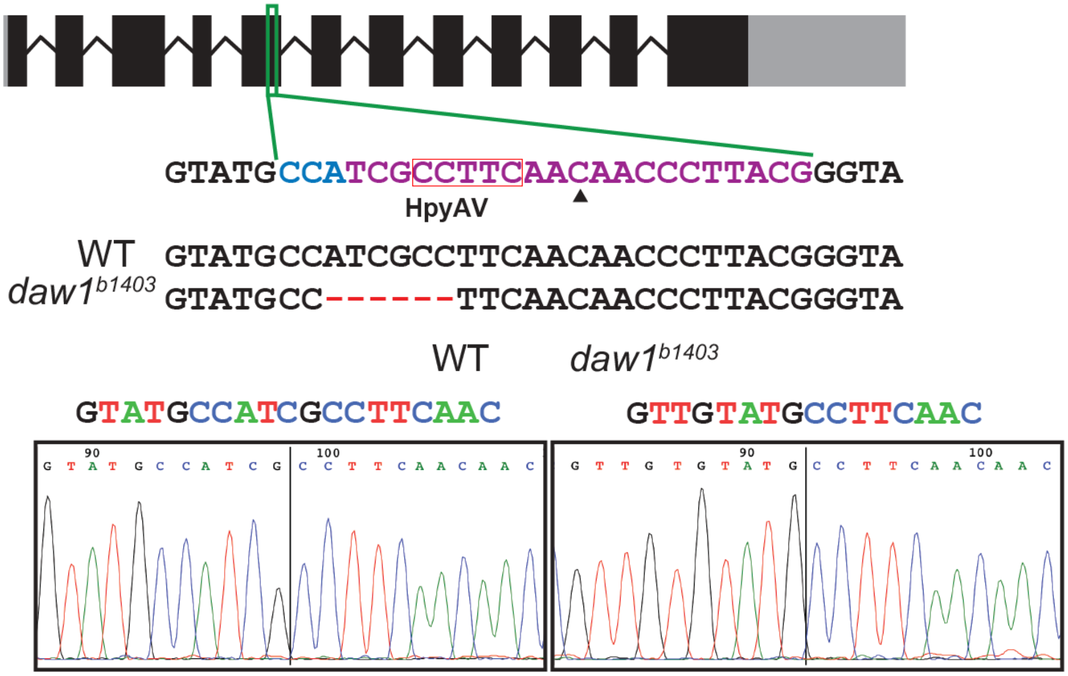

Fig. S1. Schematic of the *daw1*^{b1403} mutation. The *daw1* locus is composed of 12 exons. An sgRNA target sequence (purple) with PAM (blue) disrupted exon 5. The presence of insertion-deletion mutations was initially screened in G0 embryos by protection from restriction enzyme digestion with HpyAV. The *daw1*^{b1403} allele that was isolated encodes a 6-base pair deletion, as shown in the Sanger sequencing traces.

Development • Supplementary information

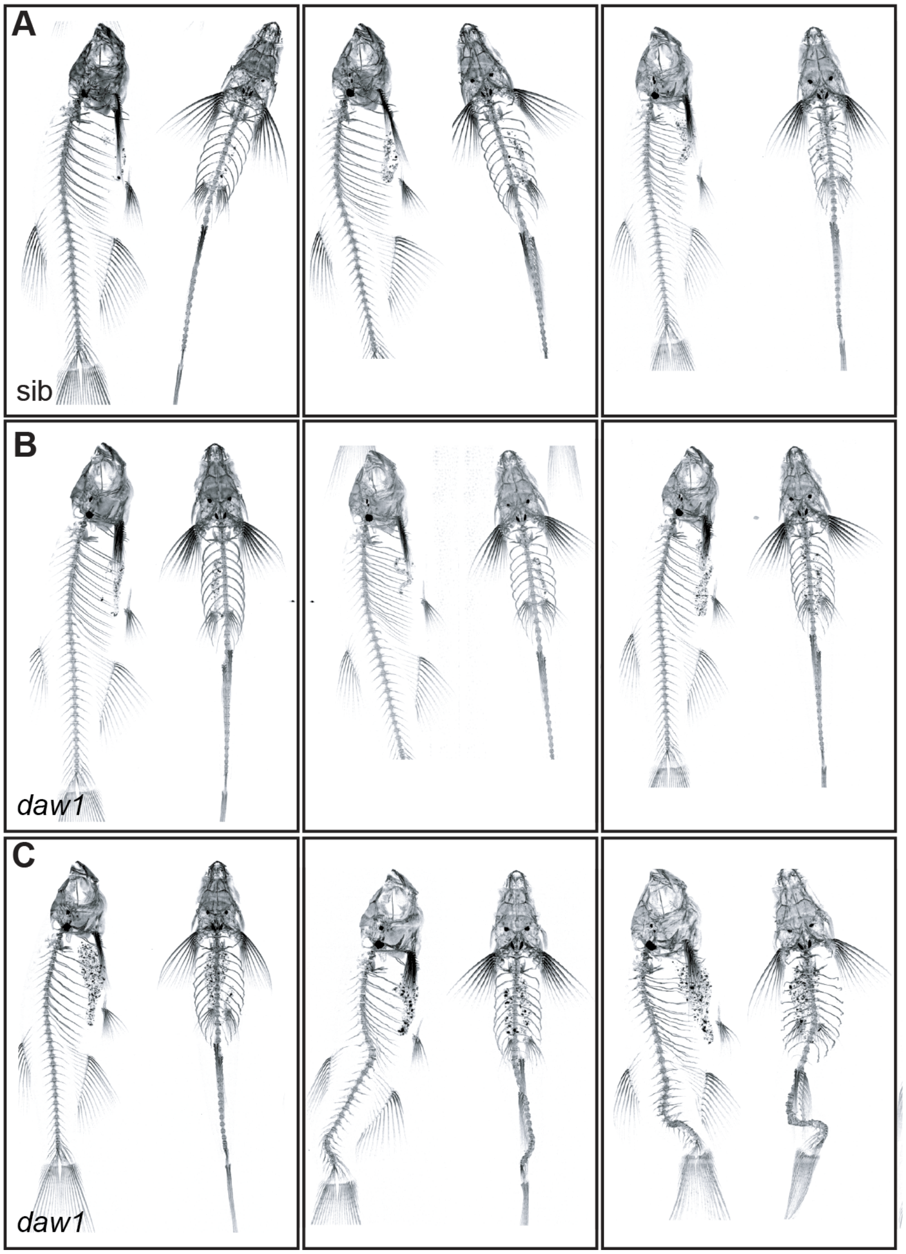

Fig. S2. µCT of *daw1*^{b1403} mutants and sibling controls. (A-C) µCT data visualized as maximum intensity projections from nine fish: *daw1*^{b1403} heterozygotes (A); *daw1*^{b1403} homozygotes that were straight at 5 d.p.f. (B); and *daw1*^{b1403} homozygotes that retained axial kinks at 5 d.p.f. (C). Spinal curves were present only in a subset of fish within the latter category (C).

Development • Supplementary information

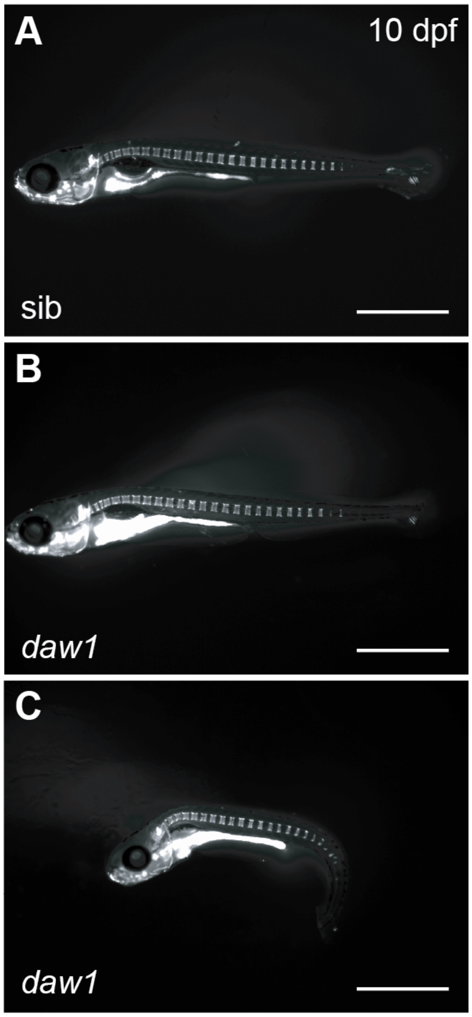

Fig. S3. Calcein staining of 10 d.p.f. *daw1^{b1403}* mutants and sibling controls. (A-C) Epifluorescent stereoscope images of *daw1^{b1403}* heterozygotes (A), and straight (B) or kinked (C) *daw1^{b1403}* homozygotes following incubation with calcein vital dye. Scale bars: A-C; 1 mm.

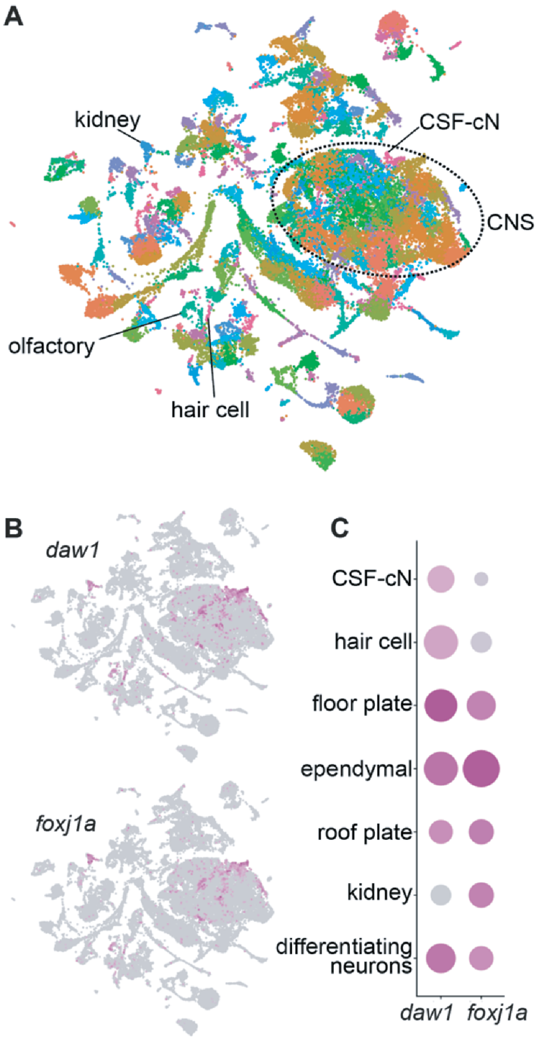

Fig. S4. Expression of *daw1* across 1-5 d.p.f. zebrafish larvae from a single-cell RNA sequencing atlas. (A) UMAP plot visualizing whole-zebrafish single cell RNA sequencing data from 1-5 d.p.f. larvae. Some clusters corresponding to cell types harboring motile cilia are highlighted. **(B-C)** UMAP (B) and dot (C) plots of *daw1* and *foxj1a* expression, showing that both expression profiles closely overlap. Foxj1a is a master regulator of motile ciliogenesis and robust marker of motile-ciliated cell types. In (C), the size of the dot indicates the number of cells expressing the gene of interest in the cluster and the intensity of purple correlates with the average number of reads within cells in the cluster. Cell types were interpreted based on expression of 16 highly expressed marker genes (Farnsworth et al., 2019). CNS – central nervous system, CSF-cN –cerebrospinal fluid-contacting neuron.

Development • Supplementary information

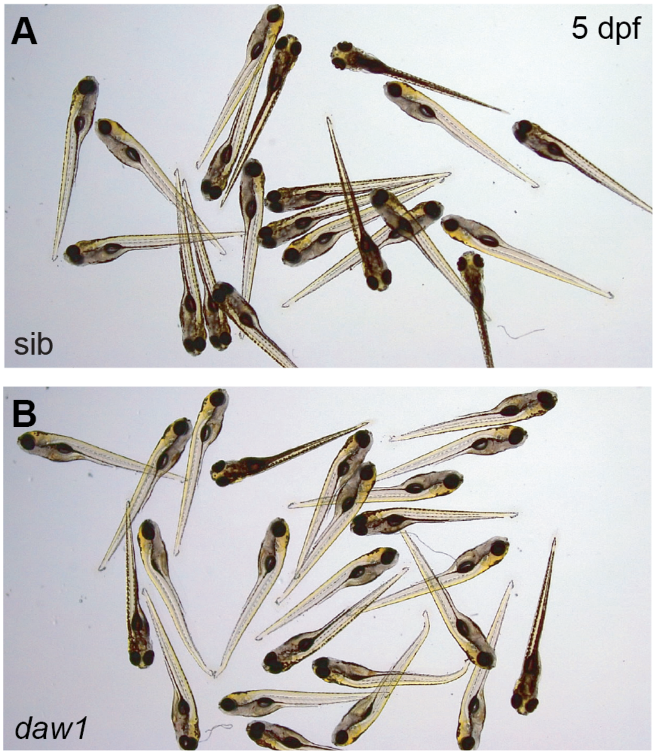

Fig. S5. Axial phenotypes in clutches of 5 d.p.f. *daw1*^{b1403} mutants. (A-B) Group shots of *daw1*^{b1403} sibling (A) and *daw1*^{b1403} mutant (B) clutches at 5 d.p.f. While most mutants successfully straighten, a subset maintain mild axial kinks.

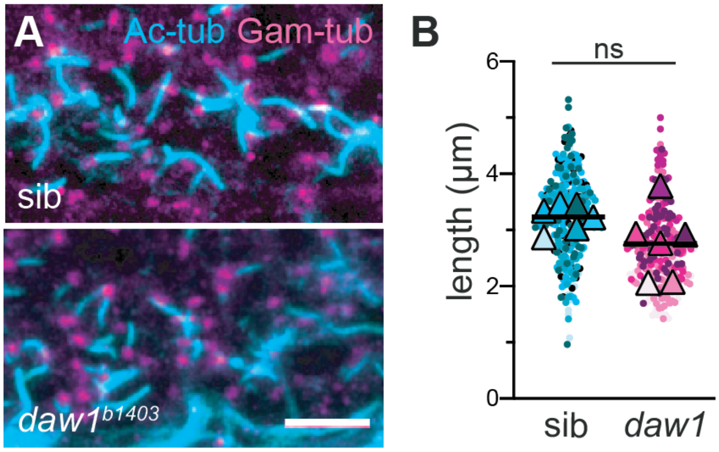

Fig. S6. Central canal cilia in *daw1^{b1403}* mutants. (A) Acetylated alpha-tubulin (cilia) and gamma-tubulin (basal body) immunolabeling of *daw1^{b1403}* siblings and homozygous mutant fish at 28 h.p.f. **(B)** Cilia length quantification reveals no significant difference between controls and mutants (*t*-test applied). Dots represent individual cilia, triangles show averages of data from different individual embryos and black lines show means. Data are from 6 siblings and mutants with >50 cilia per field of view and 3-4 fields of view per individual. ns – not significant. Scale bar: A; 5 µm.

Development • Supplementary information

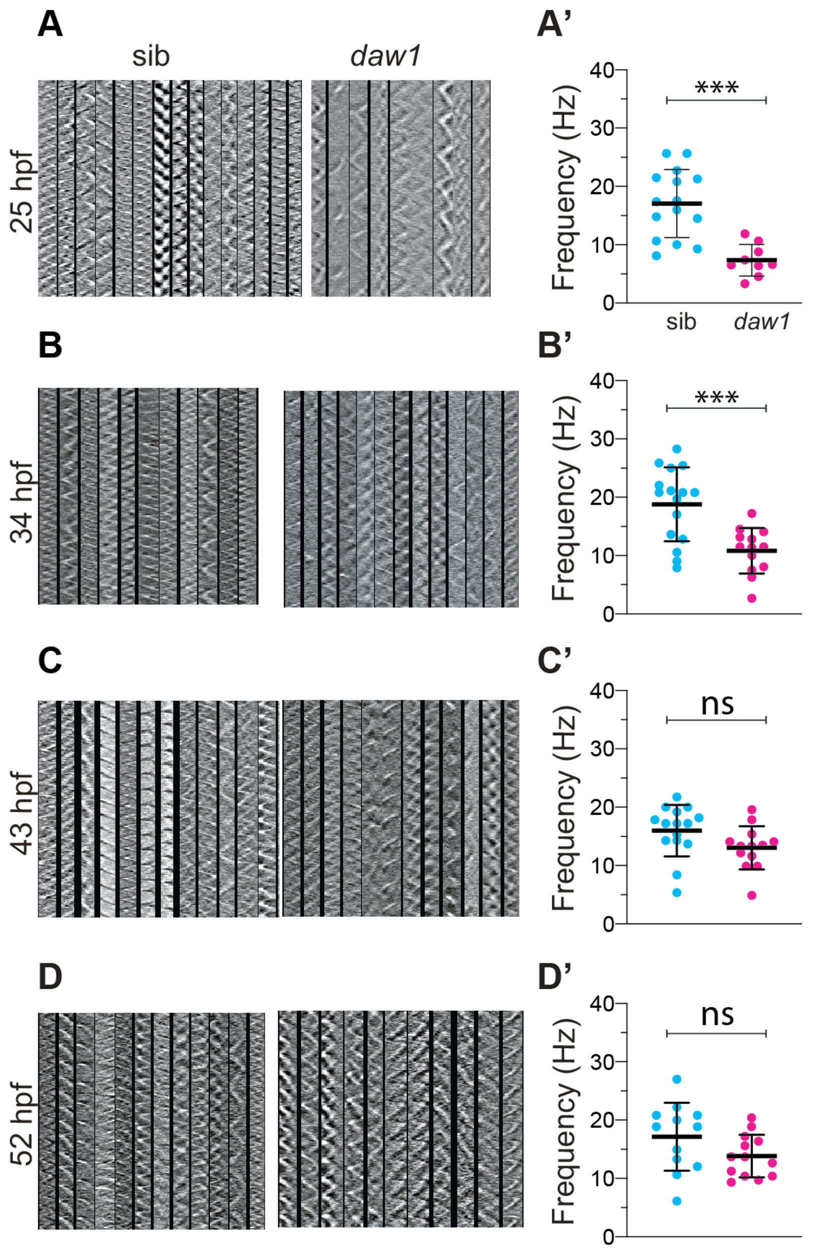

Fig. S7. Cilia kymograph and frequency data from live imaging in the central canal. (A-D) Kymographs extracted from live imaging of either *daw1*^{b1403} sibling or homozygous mutant fish at 25 h.p.f. (A), 34 h.p.f. (B), 43 h.p.f. (C) and 52 h.p.f. (D). 1 pixel height corresponds to 1 frame (4 ms); total vertical height is equal to 1 second. **(A'-D')** Quantitation of cilia beat frequencies for the subset of cilia that display any movement (Hz), extracted from kymographs. Readings are from 3-5 cilia from at least 3 regions of interest. *** $P < 0.001$, ns – not significant.

Development • Supplementary information

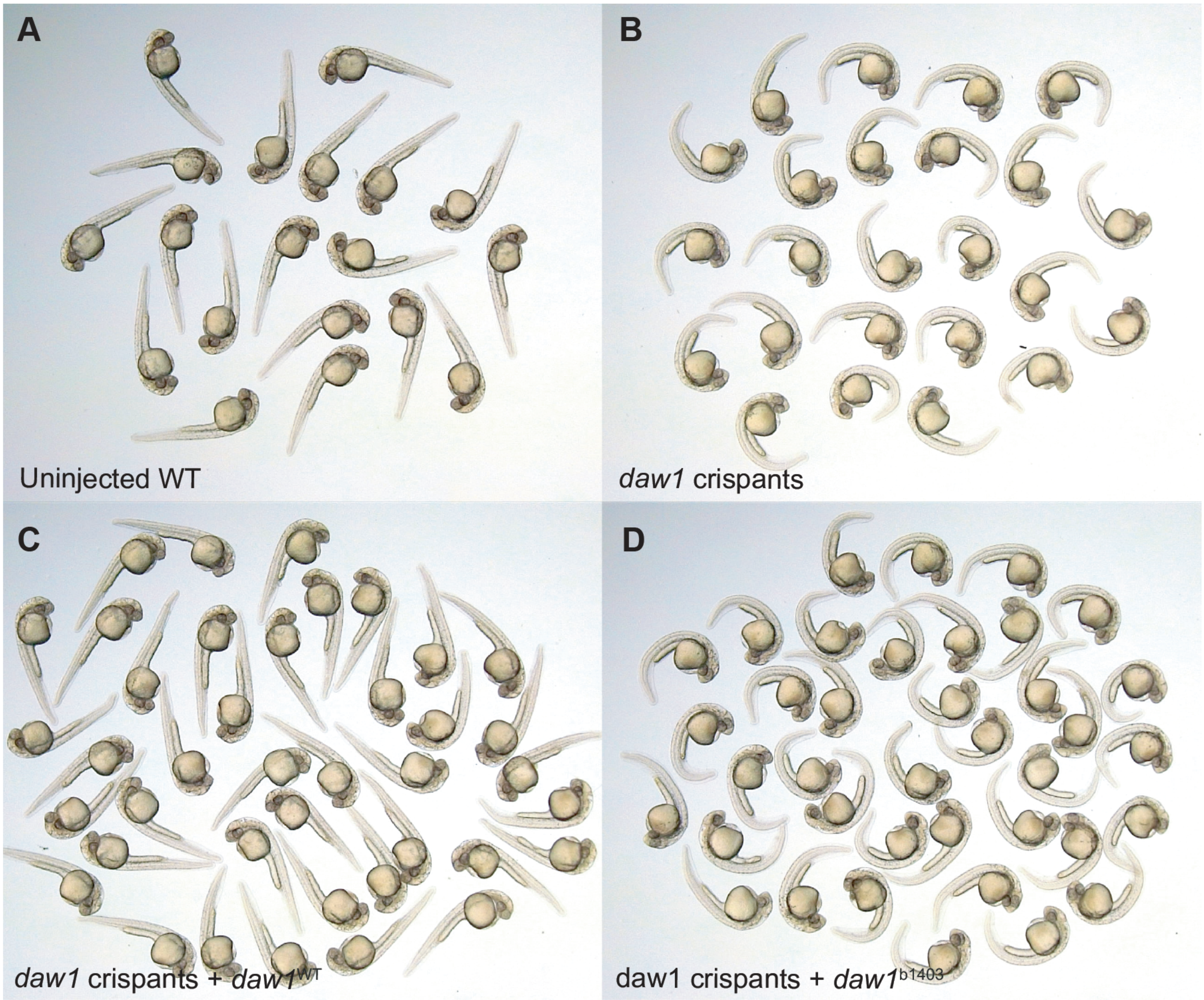

Fig. S8. *daw1* crispants phenocopy mutants and are rescued by overexpression of wild-type, but not *b1403*, *daw1* mRNA. Lateral views of 28 h.p.f. fish. Uninjected, wild-type fish (A). Crispants generated by injection of 4 sgRNAs targeting *daw1* exhibited CTD (B). Crispants were rescued by co-injected of 20 pg of wild-type *daw1* mRNA (C) but not by *daw1*^{b1403} mRNA (D).

Development • Supplementary information

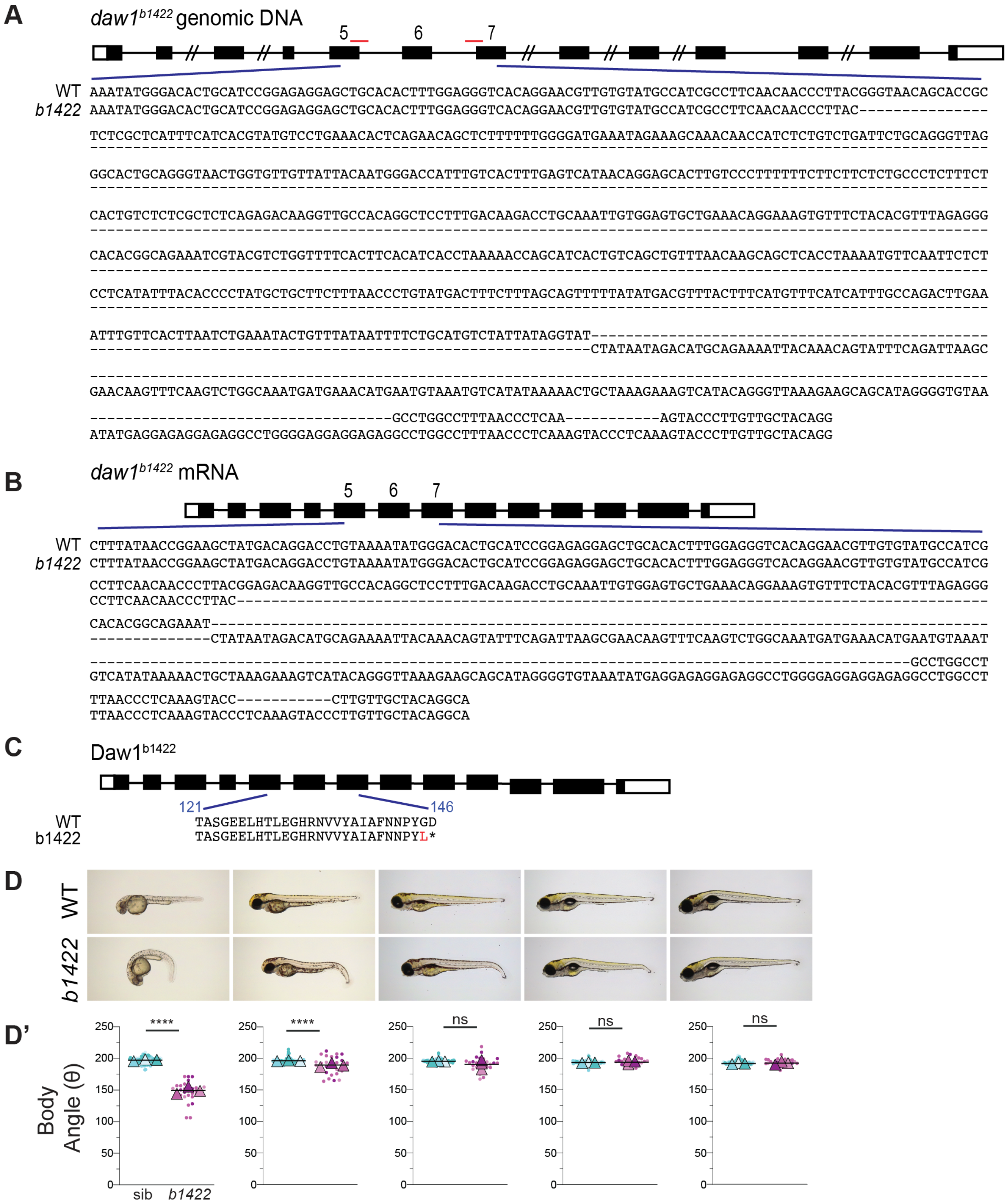

A
daw1^b1422 genomic DNA
5
6
7
WT AAATATGGGACACTGCATCCGGAGAGGAGCTGCACACTTTGGAGGGTCACAGGAACGTTGTGTATGCCATCGCCTTCAACAACCCTTACGGGTAACAGCACCGC
b1422 AAATATGGGACACTGCATCCGGAGAGGAGCTGCACACTTTGGAGGGTCACAGGAACGTTGTGTATGCCATCGCCTTCAACAACCCTTAC---------------
TCTCGCTCATTTCATCACGTATGTCCTGAAACACTCAGAACAGCTCTTTTTTGGGGATGAAATAGAAAGCAAACAACCATCTCTGTCTGATTCTGCAGGGTTAG

GGCACTGCAGGGTAACTGGTGTTGTTATTACAATGGGACCATTTGTCACTTTGAGTCATAACAGGAGCACTTGTCCCTTTTTTCTTCTTCTCTGCCCTCTTTCT

CACTGTCTCTCGCTCTCAGAGACAAGGTTGCCACAGGCTCCTTTGACAAGACCTGCAAATTGTGGAGTGCTGAAACAGGAAAGTGTTTCTACACGTTTAGAGGG

CACACGGCAGAAATCGTACGTCTGGTTTTCACTTCACATCACCTAAAAACCAGCATCACTGTCAGCTGTTTAACAAGCAGCTCACCTAAAATGTTCAATTCTCT

CCTCATATTTACACCCCTATGCTGCTTCTTTAACCCTGTATGACTTTCTTTAGCAGTTTTTATATGACGTTTACTTTCATGTTTCATCATTTGCCAGACTTGAA

ATTTGTTCACTTAATCTGAAATACTGTTTATAATTTTCTGCATGTCTATTATAGGTAT--
--CTATAATAGACATGCAGAAAATTACAAACAGTATTTCAGATTAAGC

GAACAAGTTTCAAGTCTGGCAAATGATGAAACATGAATGTAAATGTCATATAAAAACTGCTAAAGAAAGTCATACAGGGTTAAAGAAGCAGCATAGGGGTGTAA
-----------------------------------GCCTGGCCTTTAACCCTCAA-----------AGTACCCTTGTTGCTACAGG
ATATGAGGAGAGGAGAGGCCTGGGGAGGAGGAGAGGCCTGGCCTTTAACCCTCAAAGTACCCTCAAAGTACCCTTGTTGCTACAGG
B
daw1^b1422 mRNA
5
6
7
WT CTTTATAACCGGAAGCTATGACAGGACCTGTAAAATATGGGACACTGCATCCGGAGAGGAGCTGCACACTTTGGAGGGTCACAGGAACGTTGTGTATGCCATCG
b1422 CTTTATAACCGGAAGCTATGACAGGACCTGTAAAATATGGGACACTGCATCCGGAGAGGAGCTGCACACTTTGGAGGGTCACAGGAACGTTGTGTATGCCATCG
CCTTCAACAACCCTTACGGAGACAAGGTTGCCACAGGCTCCTTTGACAAGACCTGCAAATTGTGGAGTGCTGAAACAGGAAAGTGTTTCTACACGTTTAGAGGG
CCTTCAACAACCCTTAC---
CACACGGCAGAAAT--
--------------CTATAATAGACATGCAGAAAATTACAAACAGTATTTCAGATTAAGCGAACAAGTTTCAAGTCTGGCAAATGATGAAACATGAATGTAAAT
---GCCTGGCCT
GTCATATAAAAACTGCTAAAGAAAGTCATACAGGGTTAAAGAAGCAGCATAGGGGTGTAAATATGAGGAGAGGAGAGGCCTGGGGAGGAGGAGAGGCCTGGCCT
TTAACCCTCAAAGTACC-----------CTTGTTGCTACAGGCA
TTAACCCTCAAAGTACCCTCAAAGTACCCTTGTTGCTACAGGCA
C
Daw1^b1422
121
146
WT TASGEELHTLEGHRNVVYAIAFNNPYGD
b1422 TASGEELHTLEGHRNVVYAIAFNNPYL*
D
WT
b1422
D'
Body Angle (θ)
250
200
150
100
50
0
sib
b1422
****
****
ns
ns
ns

Fig. S9. ***daw1*^{b1422} mutants harbor a large deletion and premature truncation codon and phenocopy *daw1*^{b1403} mutants.** (A-C) Two sgRNAs (red) targeted the *daw1* locus. Sequencing of both DNA (A) and cDNA prepared from a mutant mRNA library (B) revealed a large deletion/ inversion leading to a premature truncation of the protein prior to most functional domains (C). (D-D') Lateral views (D) and quantitation of body curvature (D') in *daw1*^{b1422} mutants. Pairwise comparisons used two-way ANOVA adjusted for multiple comparisons. ****$P < 0.0001$, ns – not significant.

Development • Supplementary information

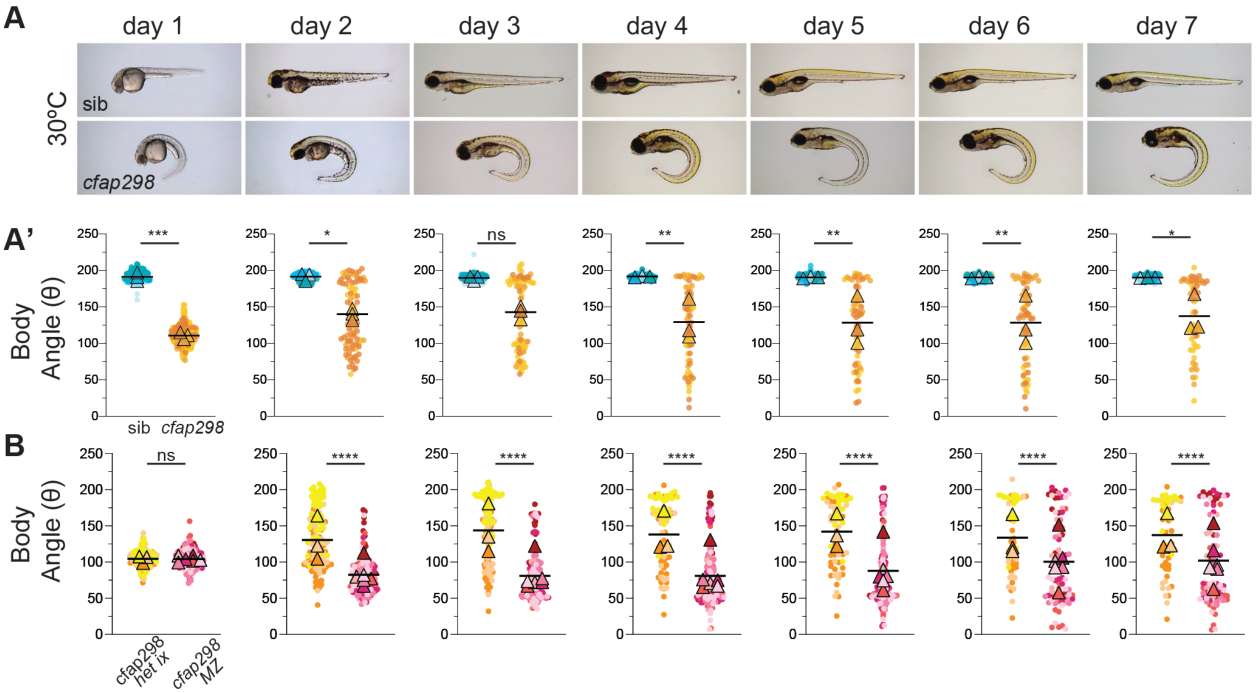

Fig. S10. Body axis curvature in *cfap298*^{tm304} mutants is temperature dependent. (A-A') Lateral views (A) and quantitation of body curvature (A') in *cfap298*^{tm304} sibling or homozygous fish over the course of 7 days of development, while housed at restrictive temperatures. **(B)** Body angle quantitation comparing zygotic and maternal-zygotic *cfap298*^{tm304} mutants. The latter undergoes lower levels of straightening, suggesting a small role for maternally-derived *cfap298* gene product. ****$P < 0.0001$, ***$P < 0.001$, *$P < 0.05$, ns – not significant.

Development • Supplementary information

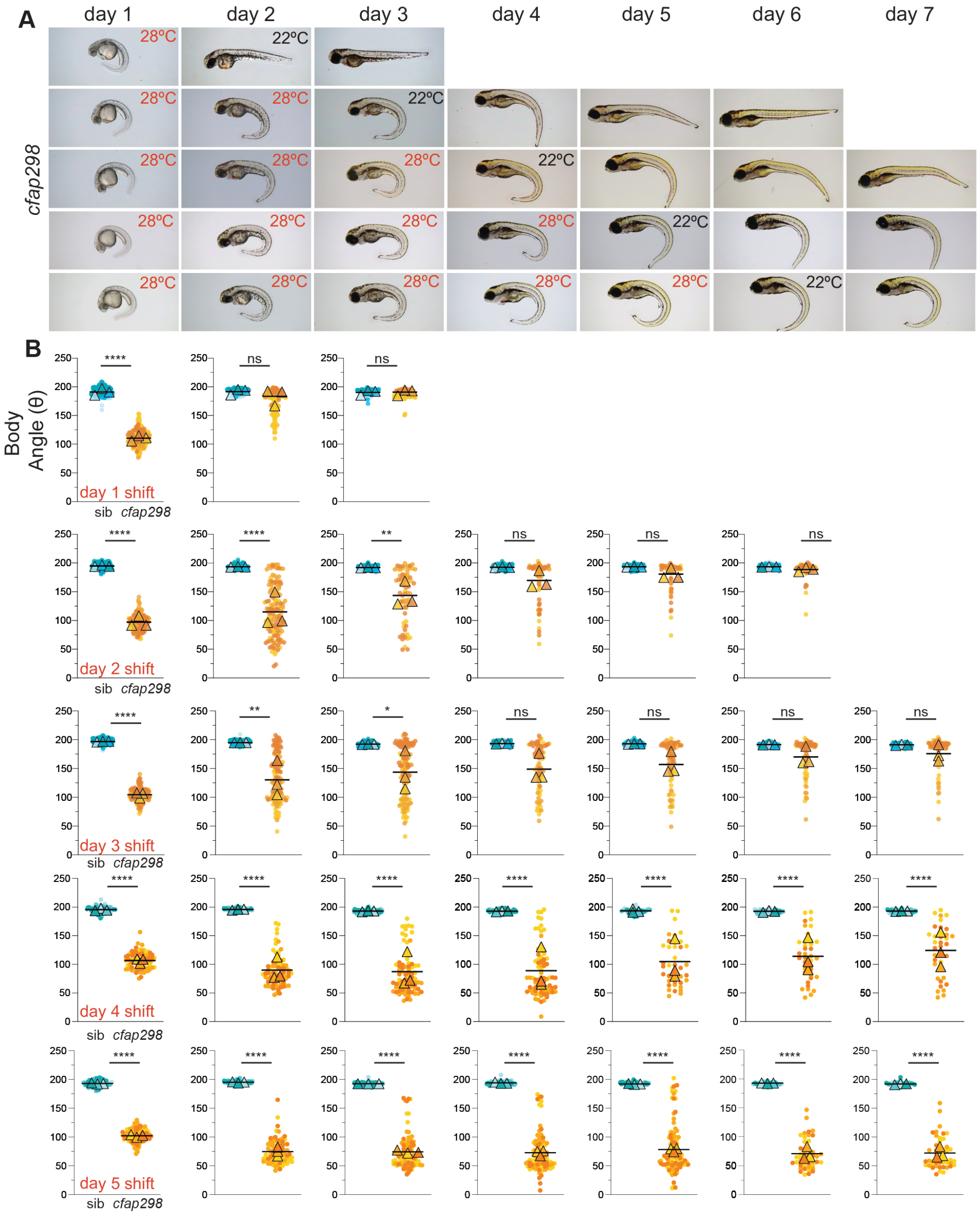

Fig. S11. Body axis recovery in *cfap298*^{tm304} mutants after temperature shifts. (A-B) Lateral views (A) and quantitation of body curvature (B) in *cfap298*^{tm304} sibling or homozygous fish over the course of 7 days of development, with temperature shifts indicated. ****$P < 0.0001$, ***$P < 0.001$, *$P < 0.05$, ns – not significant.

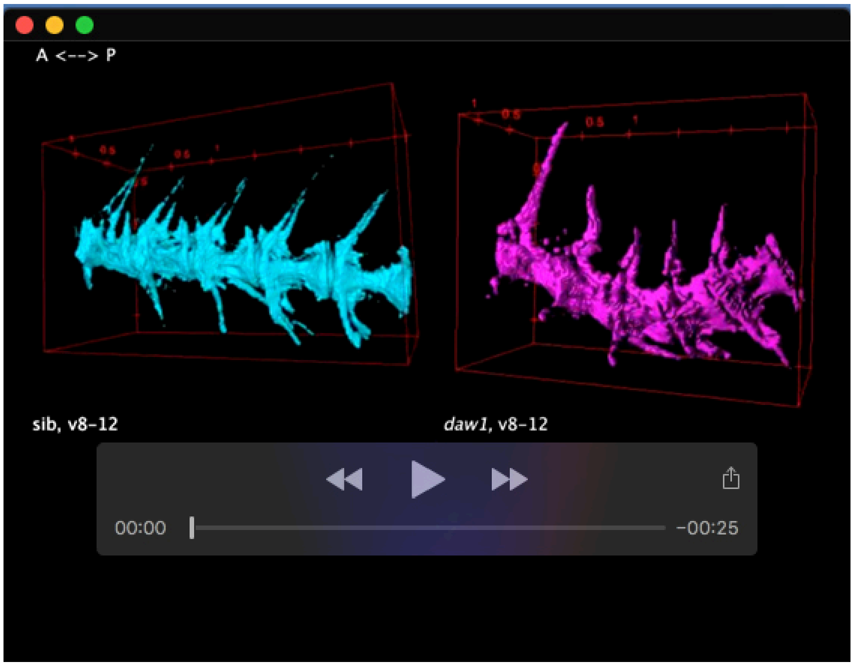

Movie 1. Reconstitutions of µCT data of sections of the vertebral column in *daw1*^{b1403} mutant adults and controls. Vertebrae 8-12 were segmented from two whole-animal µCT scans and reconstituted using 3D Viewer (ImageJ).

Development • Supplementary information

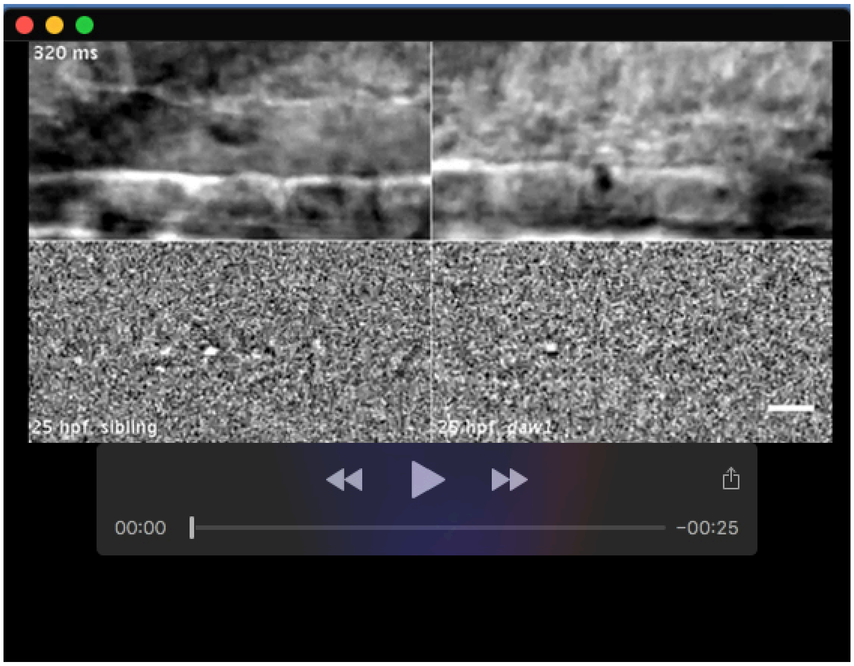

Movie 2. Central canal cilia at 25 h.p.f. in *daw1*^{b1403} mutants and controls. Images were acquired with a Nikon Ti2 inverted microscope and pco.edge sCMOS camera at 250 frames per second. The movie is replayed at 50 frames per second. Upper panels show DIC with a small Gaussian blur. Lower panels show background subtracted series using a moving average of 55 frames. Scale bar — 5 µm. Left - rostral; top - dorsal.

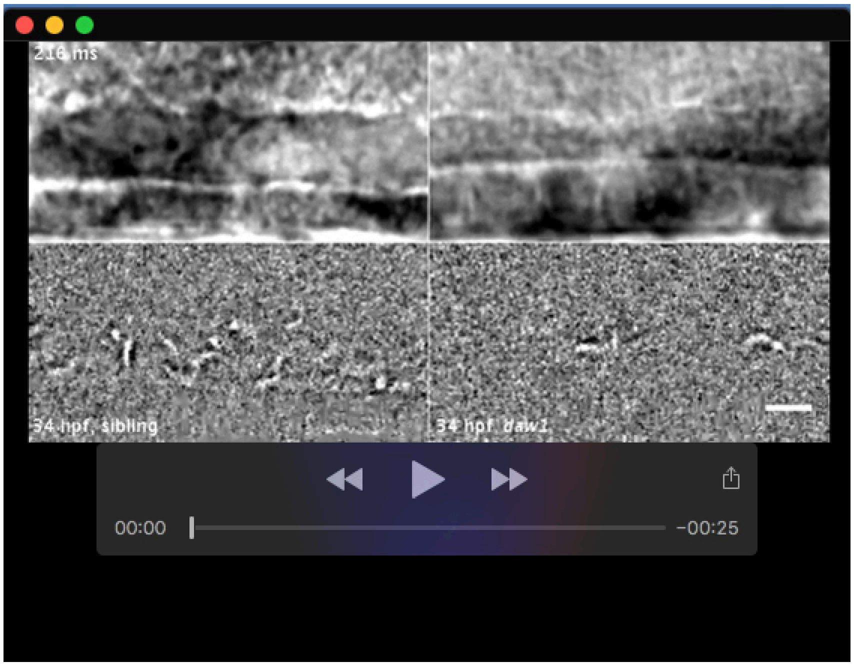

Movie 3. Central canal cilia at 34 h.p.f. in *daw1*^{b1403} mutants and controls. Images were acquired with a Nikon Ti2 inverted microscope and pco.edge sCMOS camera at 250 frames per second for 4 seconds. The movie is replayed at 50 frames per second. Upper panels show DIC with a small Gaussian blur. Lower panels show background subtracted se using a moving average of 55 frames. Scale bar — 5 µm. Left - rostral; top - dorsal.

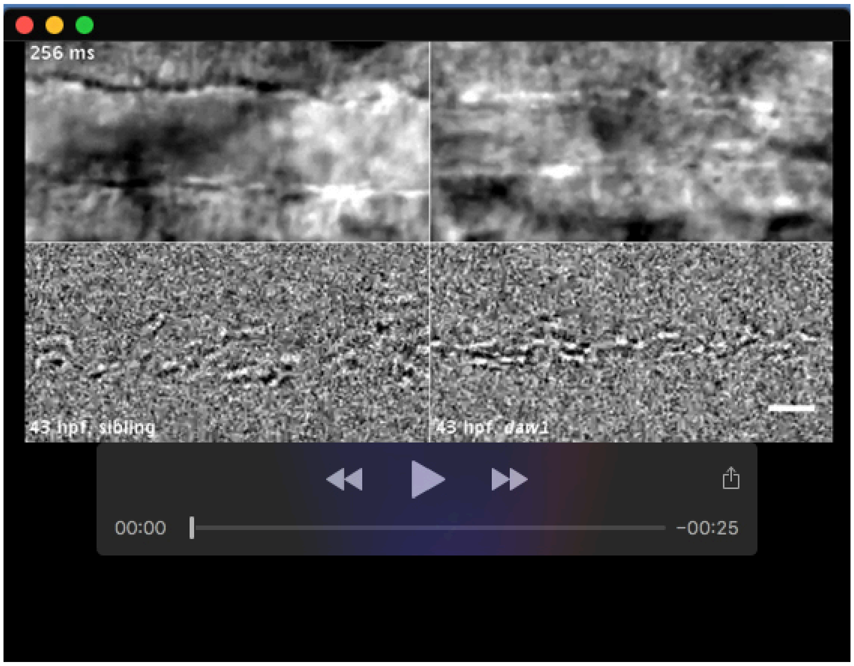

Movie 4. Central canal cilia at 43 h.p.f. in *daw1*^{b1403} mutants and controls. Images were acquired with a Nikon Ti2 inverted microscope and pco.edge sCMOS camera at 250 frames per second for 4 seconds. The movie is replayed at 50 frames per second. Upper panels show DIC with a small Gaussian blur. Lower panels show background subtracted se using a moving average of 55 frames. Scale bar — 5 µm. Left - rostral; top - dorsal.

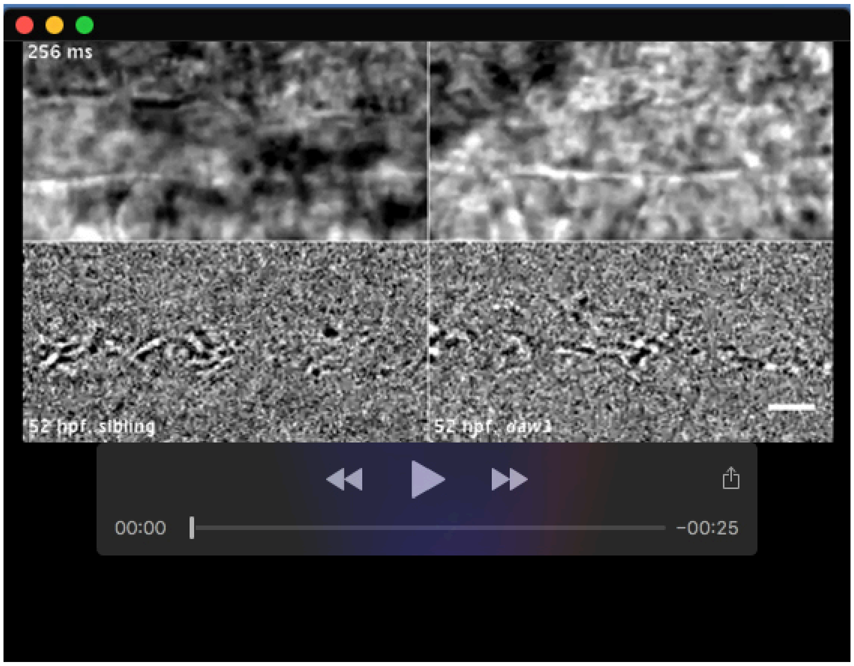

Movie 5. Central canal cilia at 52 h.p.f. in *daw1*^{b1403} mutants and controls. Images were acquired with a Nikon Ti2 inverted microscope and pco.edge sCMOS camera at 250 frames per second for 4 seconds. The movie is replayed at 50 frames per second. Upper panels show DIC with a small Gaussian blur. Lower panels show background subtracted series using a moving average of 55 frames. Scale bar — 5 µm. Left - rostral; top - dorsal.

Development • Supplementary information

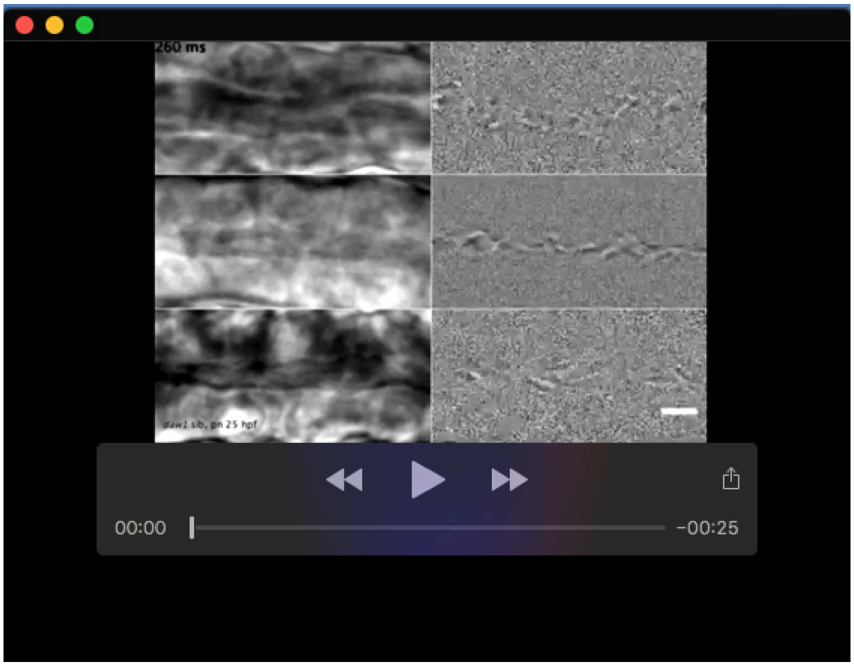

Movie 6. Pronephric duct cilia at 25 h.p.f. in control embryos. Images were acquired with a Nikon Ti2 inverted microscope and pco.edge sCMOS camera at 250 frames per second for 2 seconds. The movie (400 ms) is replayed at 50 frames per second. Left panels show DIC with a small Gaussian blur from three individual embryos. Right panels show background subtracted series using a moving average of 55 frames. Scale bar — 5 µm. Left - proximal direction; top - dorsal.

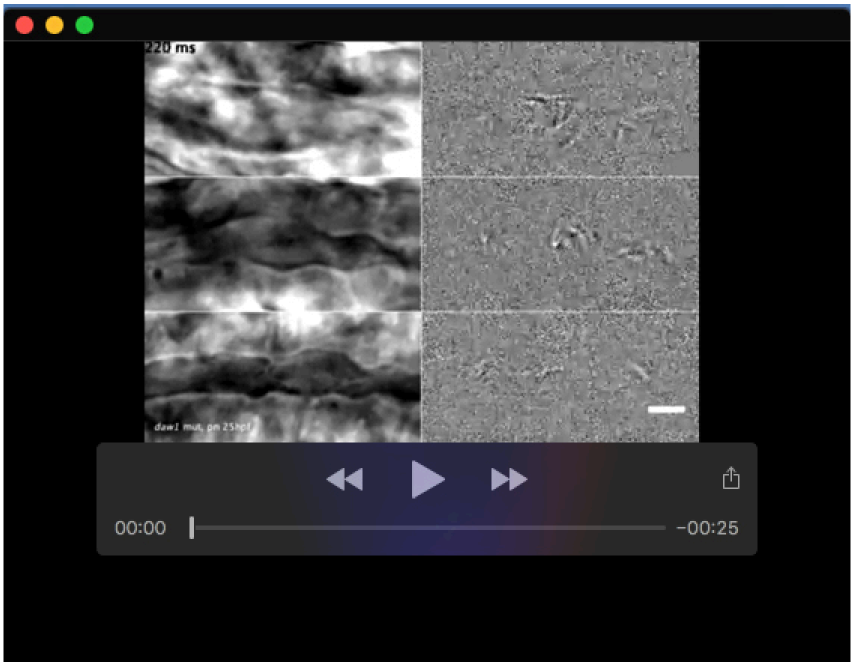

Movie 7. Pronephric duct cilia at 25 h.p.f. in *daw1^{b1403}* mutants. Images were acquired with a Nikon Ti2 inverted microscope and pco.edge sCMOS camera at 250 frames per second for 2 seconds. The movie (400 ms) is replayed at 50 frames per second. Left panels show DIC with a small Gaussian blur from three individual embryos. Right panels show background subtracted series using a moving average of 55 frames. Scale bar — 5 µm. Left - proximal direction; top - dorsal.

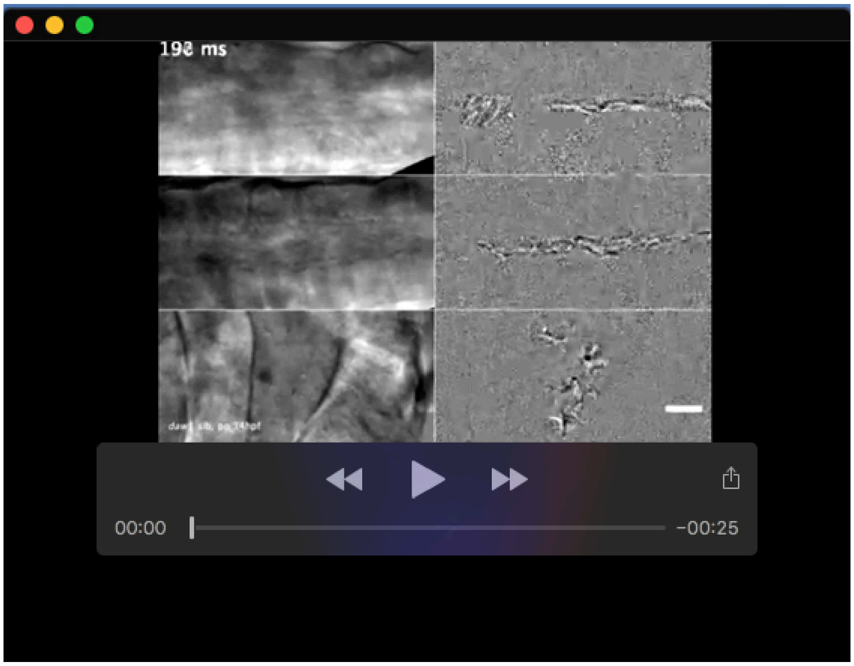

Movie 8. Pronephric duct cilia at 34 h.p.f. in control embryos. Images were acquired with a Nikon Ti2 inverted microscope and pco.edge sCMOS camera at 250 frames per second for 2 seconds. The movie (400 ms) is replayed at 50 frames per second. Left panels show DIC with a small Gaussian blur from three individual embryos. Right panels show background subtracted series using a moving average of 55 frames. Scale bar — 5 µm. Left - proximal direction; top - dorsal.

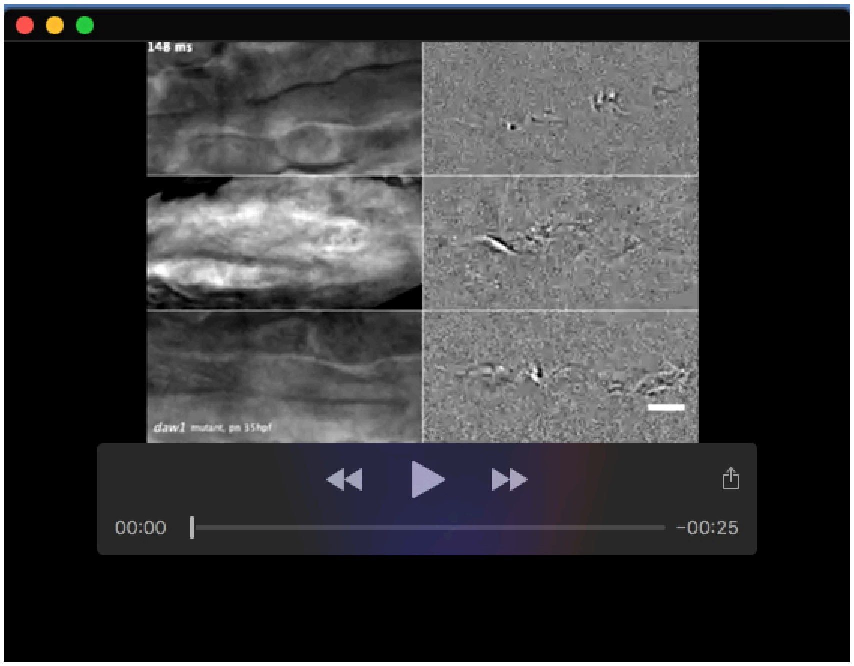

Movie 9. Pronephric duct cilia at 34 h.p.f. in *daw1^{b1403}* mutants. Images were acquired with a Nikon Ti2 inverted microscope and pco.edge sCMOS camera at 250 frames per second for 2 seconds. The movie (400 ms) is replayed at 50 frames per second. Left panels show DIC with a small Gaussian blur from three individual embryos. Right panels show background subtracted series using a moving average of 55 frames. Scale bar — 5 µm. Left - proximal direction; top - dorsal.

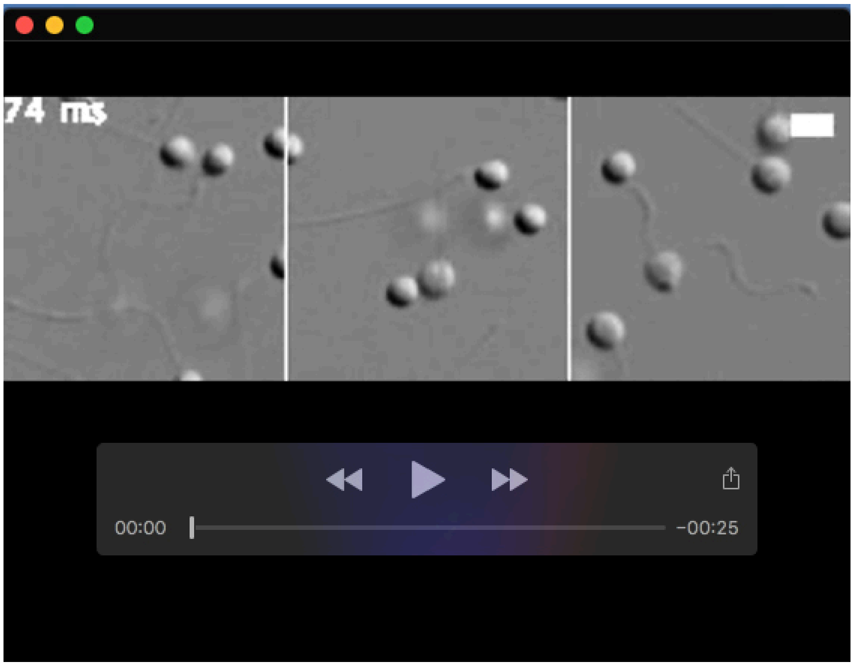

Movie 10. Sperm flagella extracted from adult male sibling controls. Images were acquired with a Nikon Ti2 inverted microscope and pco.edge sCMOS camera at 1000 frames per second for 1 second. The movie (500 ms) is replayed at 50 frames per second. Panels show data from three sperm preparations. Scale bar — 5 µm.

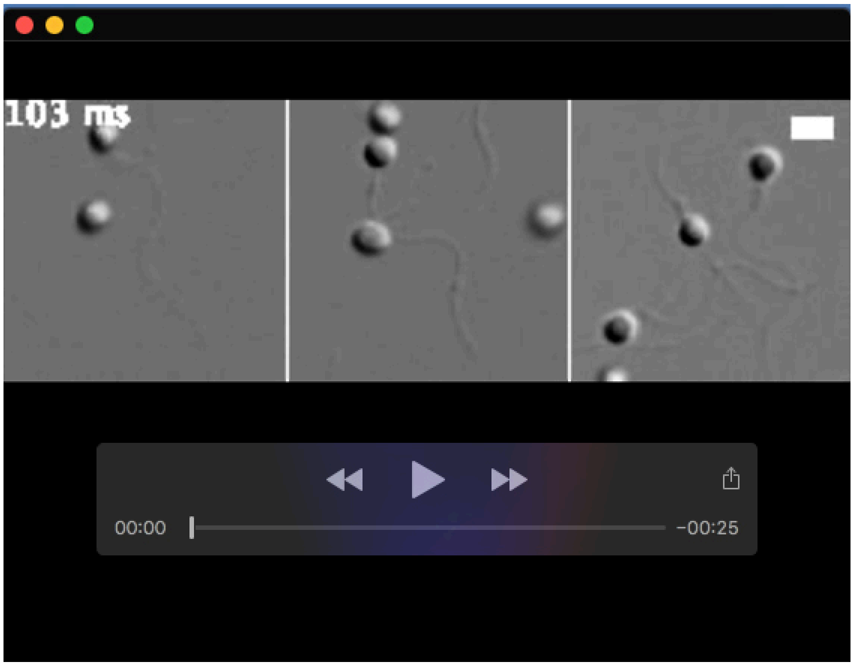

Movie 11. Sperm flagella extracted from adult male *daw1^{b1403}* mutants. Images were acquired with a Nikon Ti2 inverted microscope and pco.edge sCMOS camera at 1000 frames per second for 1 second. The movie (500 ms) is replayed at 50 frames per second. Panels show data from three sperm preparations. Scale bar — 5 µm.

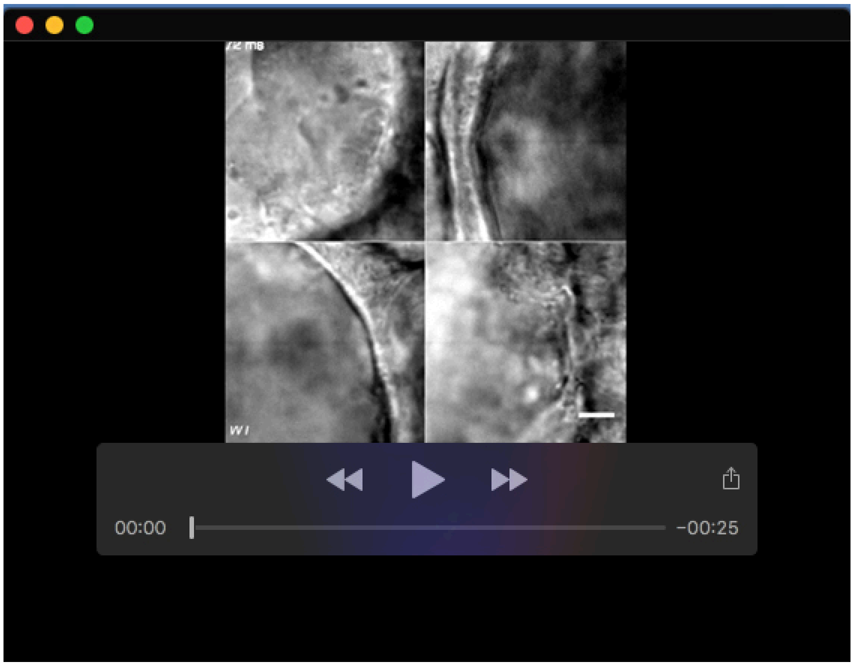

Movie 12. KV cilia in 10 somite staged wild-type control embryos. Images were acquired with a GE DeltaVision Ultra microscope at 500 frames per second for 1 second. The movie is replayed at 25 frames per second. Panels show four individual embryos. Scale bar — 5 µm.

Development • Supplementary information

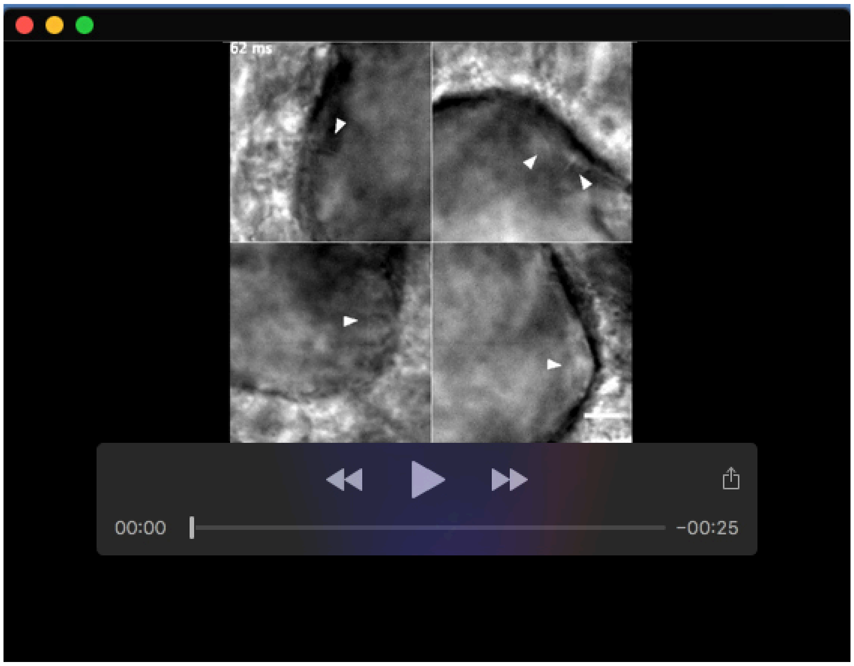

Movie 13. KV cilia in 10 somite staged *daw1*^{b1403} mutants. Images were acquired with a GE DeltaVision Ultra microscope at 500 frames per second for 1 second. The movie is replayed at 25 frames per second. Panels show four individual embryos. Arrow heads show immotile cilia. Scale bar — 5 µm.

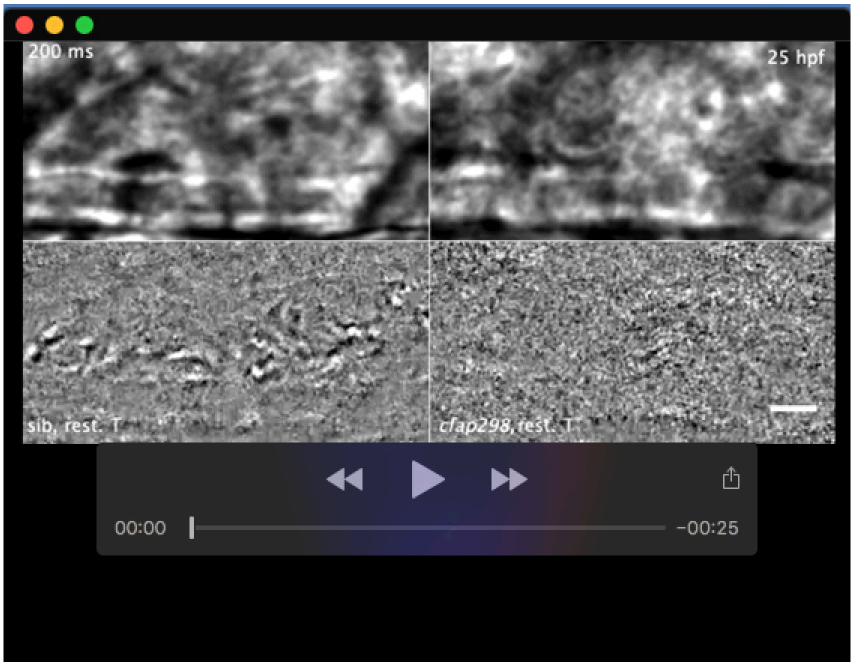

Movie 14. Central canal cilia at 25 h.p.f. in *cfap298^{tm304}* mutants and controls raised at 30°C. Images were acquired with a Nikon Ti2 inverted microscope and pco.edge sCMOS camera at 250 frames per second for 4 seconds. The movie is replayed at 50 frames per second. Upper panels show DIC with a small Gaussian blur. Lower panels show background subtracted using a moving average of 55 frames. Scale bar — 5 µm. Left - rostral; top - dorsal.

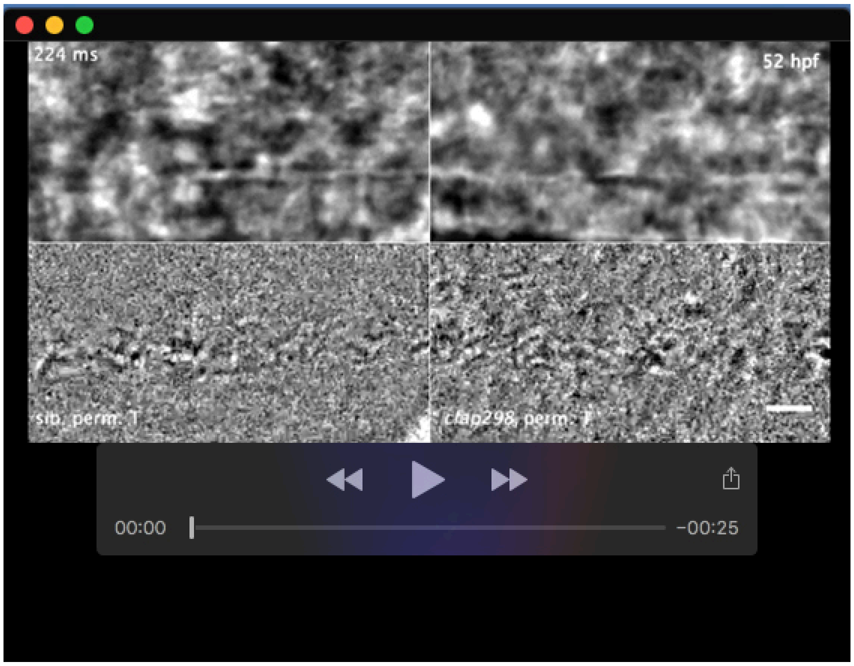

Movie 15. Central canal cilia at 52 h.p.f. in *cfap298^{tm304}* mutants and controls raised at 30°C until 28 h.p.f. then downshifted to 22°C. Images were acquired with a Nikon Ti2 inverted microscope and pco.edge sCMOS camera at 250 frames per second for 4 seconds. The movie is replayed at 50 frames per second. Upper panels show DIC with a small Gaussian blur. Lower panels show background subtracted using a moving average of 55 frames. Scale bar — 5 µm. Left - rostral; top - dorsal.

Development • Supplementary information

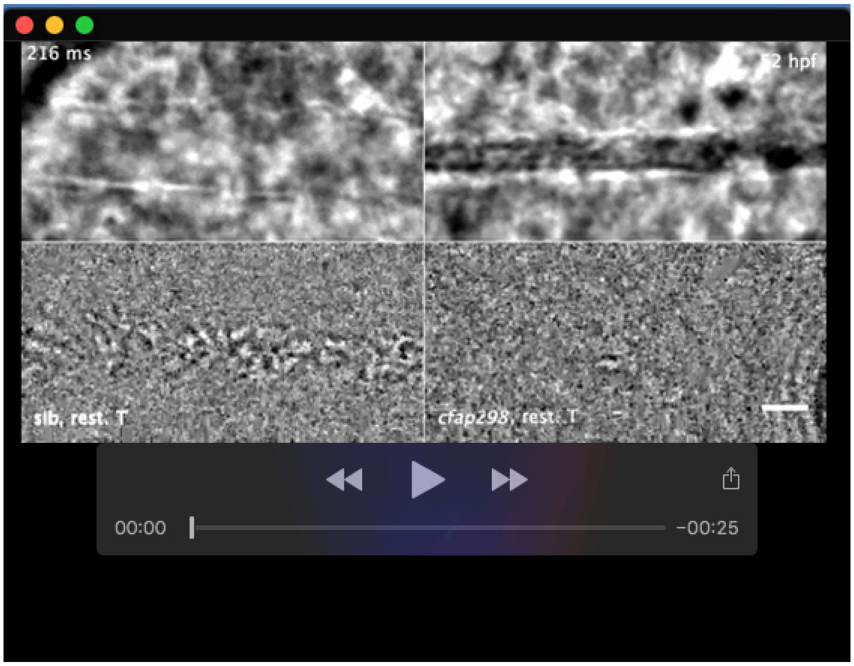

Movie 16. Central canal cilia at 52 h.p.f. in *cfap298*^{tm304} mutants and controls raised at 30°C. Images were acquired with a Nikon Ti2 inverted microscope and pco.edge sCMOS camera at 250 frames per second for 4 seconds. The movie is replayed at 50 frames per second. Upper panels show DIC with a small Gaussian blur. Lower panels show background subtracted using a moving average of 55 frames. Scale bar — 5 µm. Left - rostral; top - dorsal.
