# Supplementary material for: Daw1 regulates the timely onset of cilia motility during development
Source: Development. 2022 Jun 16;149(12):dev200017. doi: 10.1242/dev.200017 (PMC9270974; doi:10.1242/dev.200017)
Supplement: Reviewer comments [file develop-149-200017_review_history.pdf]

Daw1 regulates the timely onset of cilia motility during development

Elizabeth A. Bearce, Zoe H. Irons, Samuel B. Craig, Colin J. Kuhns, Cynthia Sabazali, Dylan R. Farnsworth, Adam C. Miller and Daniel T. Grimes

DOI: 10.1242/dev.200017

Editor: Liz Robertson

Review timeline

Original submission:	20 July 2021
Editorial decision:	26 August 2021
First revision received:	4 April 2022
Editorial decision:	29 April 2022
Second revision received:	29 April 2022
Accepted:	5 May 2022

Original submission

First decision letter

MS ID#: DEVELOP/2021/200017

MS TITLE: Daw1 Regulates the Timely Onset of Cilia Motility During Development
AUTHORS: Elizabeth A Bearce, Zoe H Irons, Samuel B Craig, Colin J Kuhns, Cynthia Sabazali, Dylan R Farnsworth, Adam C Miller, and Daniel T Grimes

Dear Dr. Grimes,

I have now received all the referees' reports on the above manuscript, and have reached a decision. The referees' comments are appended below, or you can access them online: please go to BenchPressand click on the 'Manuscripts with Decisions' queue in the Author Area.

As you will see, the referees express considerable interest in your work, but have some significant criticisms and recommend a substantial revision of your manuscript before we can consider publication. If you are able to revise the manuscript along the lines suggested, which may involve further experiments, I will be happy receive a revised version of the manuscript. Your revised paper will be re-reviewed by one or more of the original referees, and acceptance of your manuscript will depend on your addressing satisfactorily the reviewers' major concerns. Please also note that Development will normally permit only one round of major revision.

We are aware that you may be experiencing disruption to the normal running of your lab that make experimental revisions challenging. If it would be helpful, we encourage you to contact us to discuss your revision in greater detail. Please send us a point-by-point response indicating where you are able to address concerns raised (either experimentally or by changes to the text) and where you will not be able to do so within the normal timeframe of a revision. We will then provide further guidance. Please also note that we are happy to extend revision timeframes as necessary.

Please attend to all of the reviewers' comments and ensure that you clearly highlight all changes made in the revised manuscript. Please avoid using 'Tracked changes' in Word files as these are lost in PDF conversion. I should be grateful if you would also provide a point-by-point response detailing

© 2022. Published by The Company of Biologists under the terms of the Creative Commons Attribution License (https://creativecommons.org/licenses/by/4.0/).

how you have dealt with the points raised by the reviewers in the 'Response to Reviewers' box. If you do not agree with any of their criticisms or suggestions please explain clearly why this is so.

Liz Robertson
Handling Editor
Development

Reviewer 1

Advance summary and potential significance to field

This paper shows convincingly that:
1) the zebrafish homolog of ODA16, Daw1, accelerates the onset of robust cilia motility 2) delay in motility leads to body axis defects 3) remarkably, body axis curves self-correct later

The finding that zebrafish larvae possess the ability to self-correct body abnormalities is extremely important - it avails the community of a new (very powerful) model system for anatomical homeostasis, which should facilitate the study of how tissues detect large-scale defects, and correct them in reference to the species-specific target morphology. These are key open questions for developmental and regenerative biology, and the discovery of a new repair instance that can be studied using state of the art tools is a major advance.

Comments for the author

This is a very nice paper - state-of-the-art experiments, meticulously described, valuable data. Nothing more is needed to make the claims this paper makes. Thus, it can be published with only a few minor items to address:
1) "During L-R patterning, a small number of motile cilia (around 50-200) act rapidly, over a few hours (Little and Norris 2021), to initiate symmetry breaking." - this blanket statement needs to be qualified (if it is needed at all) to avoid misleading readers. There is exactly 1 paper (Nonaka et al 2002) that tests the hypothesis that cilia per se are involved in asymmetry - this is in mice (using artificial flow). All of the other studies are genetic experiments that target proteins with intracellular roles as well as ciliary ones and do not distinguish ciliary function from intracellular cytoskeletal dynamics. Also, while asymmetry might possibly be undecided as late as the appearance of the Node in mice, this is definitely not true in Xenopus and many other taxa including chick (an amniote), which already have consistent L/R identity long before cilia appear. All of this is detailed in reviews such as http://onlinelibrary.wiley.com/doi/10.1002/dvdy.22450/full . The version of this statement that would be consistent with what's actually been shown is something like "motile cilia may be involved in left-right asymmetry" (not symmetry breaking) or "break symmetry in mice". This does not diminish the paper's data or the importance thereof, and doesn't change the interpretations. It's just about accuracy and not perpetuating the "cilia are a universal symmetry break mechanism" claim (which admittedly is all over the literature) without discussion. Following this topic: are there any data on ODA16 localization or function intracellularly - is it associated with the microtubule organizing center (centrioles etc.) or affect dynein-mediated transport inside cells? That may not be known; if it's known, it should be stated for the reader. If it's not known, such roles can't be assumed not to exist.
2) I find the figure legends a bit minimal in their description - it's up to the authors and editor, but I think readers get most impact out of a paper when the figures and legends "stand alone", meaning the legend text should explain the import of what's going on a bit more (what was done and what resulted). The paper is quite concise and a bit more text in the legends could help readability and impact.
3) Figure 4 is to me the most exciting part, and I would put some labels on it to be clear what's happening. The repair of the defects is so significant, I would make sure this figure is explicit about that - maybe some sort of a timeline with a few progressive pictures of the same animal above it in larger images where it's easier to see. There are not excessive figures in this paper, so I think there's room to expand this one - take the opportunity to nail this important finding in the mind of the reader - use more space, more expressive labels to illustrate the main idea. Y axis "theta

© 2022. Published by The Company of Biologists under the terms of the Creative Commons Attribution License (https://creativecommons.org/licenses/by/4.0/).

degrees" is technically correct but how many people will skim right past this great finding if the story isn't more lavishly told here.

Reviewer 2

Advance summary and potential significance to field

The manuscript by Bearce et al. document the ciliary phenotypes of daw1 mutant zebrafish. The principal discovery from their analysis is that axial curvature of zebrafish embryos can be rescued by restoration of cilia motility at later stages of development. Although the data are well documented, there is really nothing strikingly novel. Also the paper is descriptive and there are no mechanistic insights. Furthermore, with Daw1 mutant Chlamydomonas and mice already published, the zebrafish findings presented here add marginal advance to what we already know about Daw1 function.

Comments for the author

My view is that this paper is not suitable for publication in Development as it does not provide any significant conceptual advancement.

Reviewer 3

Advance summary and potential significance to field

Ciliogenesis (the process of building cilia) often involves common molecular players and processes to assemble the diverse types of cilia found across cell types in metazoans and conserved to the unicellular protists. No protein synthesis occurs in cilia; everything must be synthesized in the cell body and imported through macromolecular machinery of intraflagellar transport (IFT) for delivery and incorporation at the growing cilia tip. In the case of motile cilia, in addition to the microtubule-based axoneme scaffold, large and complex (~1.5 MDa) molecular motors which power ciliary beat called dynein arms must also be transported en masse into assembling cilia. Likely cells have evolved players which can expedite or streamline efficient and timely delivery of these cargoes such that full cilia motility can be achieved by the cell within the developmental window for which this functionality required whether it be for mucociliary clearance of airways or movement of CSF within the brain ventricles. Daw1/Oda16/Wdr69 is one such factor first identified in C. reinhardtii as a factor involved in delivery of outer dynein arms within the axoneme via interaction with the IFT-B machinery, very much different to other primary ciliary kinesia (PCD) genes which disrupt stability or assembly of these motors. Beautiful work in several papers from the Mitchell lab showed that ODA16 appears to be needed only for efficient transport of outer arm dynein motor complexes into the flagellar compartment. They also showed that 10-20% outer arm dyneins manage to assemble in the absence of ODA16 along the length of the flagellar axoneme, leading them to hypothesize that ODA16 acts as a cofactor or adaptor that enhances the ability of the IFT machinery to transport outer arm dynein. There is a considerable body of published work on Daw1/Oda16/Wdr69 in other organisms as well including zebrafish morphants and mouse point mutants showing functional conservation where ODA import and cilia motility is impaired. What is novel in this manuscript by Bearce et al is how it tackles the temporal element of ODA import and how this alters cilia motility over time. Functional diversity of cilia in metazoa is of great interest to the field, as is how cilia can be ‘remodelled’ over time- both concepts which have long been neglected. Using zebrafish point mutants (and crispants) of daw1, the authors use beautiful whole organism as well as organelle imaging to show both physiological as well as cellular defects are differentially dependent on daw1, some of which appear to be reversible such as the body curvature defects in embryos. The authors argue that some of these gross anatomical features can ‘right’ themselves over time, whilst others that occur transiently like at KV for L/R patterning or positioning of otoliths work in ‘restricted’ developmental windows where delays cannot be tolerated. They conclude this ‘temporal restriction’ is why human DAW1 patients do not have PCD rather just laterality defects in an accompanying paper. (The paper was not shared with this manuscript). Overall the data is well presented, however it currently falls short in ‘mechanism’ without refuting possibilities for these apparently different sensitivities.

© 2022. Published by The Company of Biologists under the terms of the Creative Commons Attribution License (https://creativecommons.org/licenses/by/4.0/).

Comments for the author

Major concerns

1. Dynein import versus cilia motility phenotypes across developmental time: Again 10-20% penetrance of the CTD phenotype in mutants is the starting point and (extrapolate 10-20% ODAs in oda16 mutants in Chlamy). Were the ‘fully penetrant’ anatomical phenotypes in the first day (otoliths and heart looping) because there was no ODAs imported (delayed) thus no motility at all in these windows, or was there some motility (10-20%), but it was not enough (physiological differences in requirements for motility)? 8 somite stage of KV cilia mostly motile in controls, what about your oda16 mutant and if not motile, how late can you see if KV cilia start to move? Is it time that is the issue or sensitivity to cilia movement (force and frequency)? Alternately could there be tissue-specific compensation in some cilia types by another factor? How long lived are cilia/ciliated cells in the central canal? The ‘downshift’ of the cfap298 ts allele goes some way to address this but the latest timepoint 3dpf could still be developmental plasticity- taking it out to a further timepoint would go a long way to address this. What is the latest point you could downshift at to restore body curvature? Based on your Figure 2 F’-I’ it looks like cilia beat frequency/ coverage- (a term which needs better explanation- frequency and waveform may be more apropos in the field, as we know these parameters change over time in motile cilia development and coverage would like only capture how wide a cilia extends to cover the central canal as opposed beat frequency and changes in waveform to more synchronized) is still emerging in controls at your last time point in your downshift assays but does it continue to increase and/or mature so there less spread of the distributions?

- Recommendations: Live imaging of KV cilia in controls and mutants- is motility delayed (or never starts). Clearer use of cilia beat frequency and waveform, density may not capture changes.

2. Citations: There is lots of exciting things in this paper but he narrative of the paper needs to be much more transparent about all that was known about ODA16/DAW1 previously, shaped around what was not known rather than present findings that support the studies that came before. For example, Oda16/Wdr69 mutants and morphants are shown not to affect cilia length and number in fish (Gao et al 2010), planaria (Lesko et al 2020), mice (Solomon et al 2017) or Chlamy (Ahmad et al 2008) but in the 6th paragraph of the results discussion section, authors focus on the IFT46 interaction (citing the Ahmad paper) and IFT46 mutations causing shortened cilia. There is no evidence that ODA16 disrupt anterograde IFT from any of these previous studies and this line of analysis seems tangential. (And a missed talking point Esben's new paper showing differences in human and Chlamy ODA16 structure where the N-terminus that interacts with IFT46 is not conserved, other adaptors for ODA import likely to exist, possible differences between species. https://www.ncbi.nlm.nih.gov/pmc/articles/PMC7255517/) It is not until the 8th paragraph that this role for ODA16/WDR69 is discussed in more detail- this all needs to be at the front of this manuscript much more than it currently is in the introduction. Similarly, when talking about allelism in paragraph 9, the fact that in all these other systems 10-20% of ODAs still exist suggest it is required but not essential for the process and that similarly daw1b1403 is a strong loss-of-function allele, I would take out the ‘null condition’ as it does not add more than the previous term.

- Recommendations: Textual edits and narrative overhauls.

3. Differences in cilia types and cellular sensitivities to dysfunction: It seems key to your central tenet that longer lived cilia (on longer lived cells) can improve their motility over time, whilst transient cilia (or on transient cell types) are not given the opportunity to catch up their dynein quotient necessary for ‘normal’ motility. The Daw1 mice showed significant mucociliary clearance defects which suggest that while motility grossly is normal (i.e. ciliary beat frequency), the necessary force generated by ODAs is subpar. Importantly they do TEM to show partial loss of ODAs in these cells, explaining this phenotype. This is what is lacking in my opinion in the paper by Bearce currently- is the content of the ODAs shifting over time, slow to accumulate on the mutant axoneme and thus slow, dyskinetic movement? Is the rate the same between diverse cilia/cell types (ei KVs versus central canal cells), just how permanent a structure it is different (i.e. longer time to catch up)?

- Recommendations: This is about import of ODAs into cilia- IF staining of KV cilia versus different timepoints in central canal, or TEM sections to show content of ODAs changing over time in different cilia types.

© 2022. Published by The Company of Biologists under the terms of the Creative Commons Attribution License (https://creativecommons.org/licenses/by/4.0/).

Minor concerns
1. Improved scholarship in introduction of cilia motility- cilia motility is powered by dynein arms which come in two flavours outer (ODAs) and inner (IDAs) which power cilia beat frequency and waveform respectively, in the abstract and introduction.
2. Abstract: change ‘accelerates’ to ‘facilitates’. Accelerates would suggest making it faster than normal and those experiments were not done. Consider changing ‘which sets the time’ to ‘promotes the onset of timely cilia motility’? Again, ‘setting the time’ is usually terminology for clock or pacemaker type functions, which this is not. Last intro paragraph ‘instead speeds us the rate at which’ consider ‘instead ensures timely and robust motility is achieved’.
3. Second paragraph be specific about which species you are referring to. Cilia on multiciliated cells do not turn over gradually but the cells of those epithelia do (this comes up again in the results section). You are missing the airway cell paper Oltean paper from the Brody lab on how cilia are build, motors move in over time and beat changes- https://pubmed.ncbi.nlm.nih.gov/29851510/.
4. Results section: ‘viable and fertile’- there are no sperm defects in the mutants- no flagellar motility defects and if so, why not?
5. ‘At 25 hpf, we found disruptions to otolith positioning’ should explicitly state confirming the results of the Gao paper, similar with the L/R paper.
6. Daw1 mice have significant mucociliary clearance defects, as well as high incidence of hydrocephaly supports different tissue sensitivities to cilia motility (onset) but also quality.
7. Suggestion to discuss PCD more as a spectrum of cilia motility defects, not all tissues equally affected would be useful when you discuss your human data.
8. Figure 1C-E: consider annotating what the difference is from D and E on the figure more clearly. Consider maybe adding percentages?
9. Figure 2F- does not show the frequency as stated in the text- this data is important. Explain what ‘cilia density’ is in the legend. The results section for this figure mentions small number of cilia beating slowly and erratically but having this data in the figure would be helpful.
10. Figure S4: Kidney is labelled in both daw1 and foxj1a(B) but C shows no kidney expression for daw1. Maybe explain the ‘differentiating neurons’ track given this legend is specifically about motile cilia genes.
11. Figure S6: how many animals were looked at per genotype in legend.
12. Figure S7. Label your time points on the figure. What does each point refer to- one cilia frequency reading per animal or independent readings from the same animal (how many animals were looked at)?
13. Figure S9A’: Even at the non-permissive temperature a sizeable promotion of cfap298 mutants seem to catchup within a day, with this huge distribution (tail) by day 4. Are the escapers such that this is a leaky allele? Are their cilia immotile? Is this seen in your other cfap allele Figure 3? This difference is never commented on and from the M&M section they seem to be the same allele?
14. The last sentence in the results discussion section is challenging to comment on the validity as accompanying (submitted) manuscript was not submitted to allow review. This is unusual.

Textual edits
1. Introduction, 1st paragraph: add ‘motility to rescue CTD phenotypes’ and this sentence could benefit from a little edit for clarity, maybe with ‘embryonic rescued mutants going on to develop spinal curves’?
2. Throughout- idiopathic scoliosis should be lower case throughout the manuscript.
3. Remove par excellence?
4. IFT should be lower case intraflagellar transport.

© 2022. Published by The Company of Biologists under the terms of the Creative Commons Attribution License (https://creativecommons.org/licenses/by/4.0/).

First revision

Author response to reviewers' comments

Daw1 Peer Review Response

We thank all three reviewers for taking the time to read our paper and provide us with feedback. We were pleased that reviewer 1 considered our work a "major advance" with "state-of-the-art experiments, meticulously described" while reviewer 3 noted that the data were "well presented" and "novel [in how it] tackles the temporal element of ODA import".

Over the last few months, we have worked hard to improve our manuscript in line with reviewer suggestions.

Here, we include a point-by-point response to the review comments. To summarize the major improvements:

1. We now include a new *daw1* mutant line which harbors a large deletion (all of exon 6) and an insertion. cDNA sequencing showed that the resulting transcript harbors a domain deletion and multiple premature truncation codons. Like our previously described 2-amino acid deletion line and crispants, homozygous mutants for this new line displayed fully penetrant ventral curves at 1 dpf which self-corrected over time. This now definitively demonstrates that recovery from ventral curves occurs in a *daw1* loss-of-function condition. We describe these new results, including allele generation, DNA and cDNA sequencing, and time courses of spinal curve recovery in **Supplementary Figure 9**.
2. While our initial manuscript focused on cilia in the central canal, we have now expanded our analysis to KV cilia, kidney cilia (at more than one time point) and the sperm flagellum. Our results indicate that KV cilia are entirely immotile in *daw1* mutants, the sperm flagellum is minimally impacted, while pronephric cilia display delay in achieving robust beating and bundling. This reinforces our idea that Daw1 function enhances the time at which robust beating is achieved in embryonic cilia. Since KV cilia are short-lived, we suggest that Daw1 function is essential for allowing KV cilia to rapidly achieve robust beating. In agreement, *daw1* mutants showed severe L-R patterning defects.
3. We extended our *cfap298* downshift experiments to 5 dpf as suggested. We found that the later cilia were reactivated by temperature downshift, the longer it took for body curves to correct. When shifting at 5 dpf, curves were not able to correct by 7 dpf, the latest time point we could assess as fish could not survive beyond this point with body curves.
4. We have altered our narrative in line with the suggestions of reviewers. Importantly, we also emphasize the potential of cell-type specific differences in Daw1 function and requirement.

Reviewer 1

Advance Summary and Potential Significance to Field:

This paper shows convincingly that:

1) the zebrafish homolog of ODA16, Daw1, accelerates the onset of robust cilia motility
2) delay in motility leads to body axis defects
3) remarkably, body axis curves self-correct later

The finding that zebrafish larvae possess the ability to self-correct body abnormalities is extremely important - it avails the community of a new (very powerful) model system for anatomical homeostasis, which should facilitate the study of how tissues detect large-scale defects, and correct them in reference to the species-specific target morphology. These are key open questions for developmental and regenerative biology, and the discovery of a new repair instance that can be studied using state of the art tools is a major advance.

Comments for the Author:

This is a very nice paper - state-of-the-art experiments, meticulously described, valuable data. Nothing more is needed to make the claims this paper makes. Thus, it can be published with only a few minor items to address:

1) "During L-R patterning, a small number of motile cilia (around 50-200) act rapidly, over a few

© 2022. Published by The Company of Biologists under the terms of the Creative Commons Attribution License (https://creativecommons.org/licenses/by/4.0/).

hours (Little and Norris 2021), to initiate symmetry breaking." - this blanket statement needs to be qualified (if it is needed at all) to avoid misleading readers. There is exactly 1 paper (Nonaka et al 2002) that tests the hypothesis that cilia per se are involved in asymmetry - this is in mice (using artificial flow). All of the other studies are genetic experiments that target proteins with intracellular roles as well as ciliary ones and do not distinguish ciliary function from intracellular cytoskeletal dynamics. Also, while asymmetry might possibly be undecided as late as the appearance of the Node in mice, this is definitely not true in Xenopus and many other taxa including chick (an amniote), which already have consistent L/R identity long before cilia appear. All of this is detailed in reviews such as http://onlinelibrary.wiley.com/doi/10.1002/dvdy.22450/full. The version of this statement that would be consistent with what's actually been shown is something like "motile cilia may be involved in left-right asymmetry" (not symmetry breaking) or "break symmetry in mice". This does not diminish the paper's data or the importance thereof, and doesn't change the interpretations. It's just about accuracy and not perpetuating the "cilia are a universal symmetry break mechanism" claim (which admittedly is all over the literature) without discussion. Following this topic: are there any data on ODA16 localization or function intracellularly - is it associated with the microtubule organizing center (centrioles etc.) or affect dynein-mediated transport inside cells? That may not be known; if it's known, it should be stated for the reader. If it's not known, such roles can't be assumed not to exist.

We agree that cilia are likely not the essential starting point of symmetry breaking in most organisms. Indeed, cilia are clearly not involved in invertebrates and some vertebrates, while they may be part of an early "asymmetry amplification" mechanism in other vertebrates. As such, we have toned down our reference to the centrality of cilia in breaking symmetry. We changed motile cilia are "required for" to "involved in" left-right asymmetry in paragraph 1. In paragraph 2, we removed our comment that cilia "initiate symmetry breaking" so that the sentence now only reads as cilia acting "During L-R patterning".

The current model for the function of Daw1 intracellularly involves the control of import of ODAs into the cilium, with Daw1 acting as a bridge between IFT particles and ODAs. This is largely based on protein- protein physical interaction and genetic experiments. Daw1 sub-cellular localization is not known. As such, we expressed mCherry-tagged wild-type Daw1 and imaged its sub-cellular localization in the pronephric duct, a motile-ciliated tissue (**Figure A** - for reviewer only). We saw cytosolic expression (broadly) including some bright puncta, which could potentially be phase-separated DynAPs (Huizar et al., 2018), though we lack sufficient evidence to make this claim strongly. We do not believe these results are sufficient to include in the manuscript at this time because this was performed with overexpression constructs. We plan to continue this line of investigation by generating Daw1-specific antibodies, but we suggest that a full description of Daw1 sub-cellular localization is beyond the scope of the current manuscript.

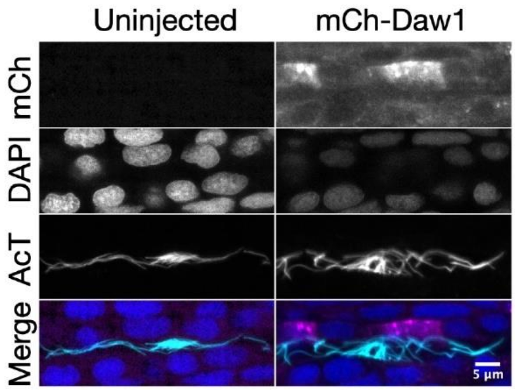

Figure A: Daw1 sub-cellular localization. After overexpression of an mCherry (mCh)-tagged Daw1, we observed Daw1 expression in cytoplasmic puncta in pronephric duct multi-ciliated cells. AcT - acetylated alpha-tubulin.

2) I find the figure legends a bit minimal in their description - it's up to the authors and editor, but

© 2022. Published by The Company of Biologists under the terms of the Creative Commons Attribution License (https://creativecommons.org/licenses/by/4.0/).

I think readers get most impact out of a paper when the figures and legends "stand alone", meaning the legend text should explain the import of what's going on a bit more (what was done and what resulted). The paper is quite concise and a bit more text in the legends could help readability and impact.

Our figure legends were originally more expansive but we had to cut them to stay within the word limit of the article type. We have have now gone back and tried to make the figure legends more descriptive in places, but they nevertheless remain short due to the tight limits on word count for this article type.

3) Figure 4 is to me the most exciting part, and I would put some labels on it to be clear what's happening. The repair of the defects is so significant, I would make sure this figure is explicit about that
- maybe some sort of a timeline with a few progressive pictures of the same animal above it in larger images where it's easier to see. There are not excessive figures in this paper, so I think there's room to expand this one - take the opportunity to nail this important finding in the mind of the reader - use more space, more expressive labels to illustrate the main idea. Y axis "theta degrees" is technically correct but how many people will skim right past this great finding if the story isn't more lavishly told here.

We thank the reviewer for the advice in making Figure 4 more impactful. We have made the following changes:

A) We've changed the axis label to read as "Body angle" rather than "theta degrees".

B) We have extended the time course to temperature shifts at 5 dpf and imaging to 7 dpf (after which, the fish start to die due to inability to feed when curved). These new data are included in the new Figure. To summarize, the later we shift the temperature and activate cilia motility, the more slowly and less completely recovery from body curves occurs.
C) We have included a new graphical depiction of the finding in Part F. The idea here is to summarize the various conditions (wild-type, *daw1* mutant, and *cfap298* mutants at increasing ages of temperature shift) at different positions on a sundial - the further around the sundial, the later robust cilia beating is achieved during development in the central canal. The effects of these differential times are then depicted by the silhouettes of zebrafish, showing that straightening occurs in these conditions but is delayed by increasing amounts the later cilia motility onsets.

New Figure 4:

© 2022. Published by The Company of Biologists under the terms of the Creative Commons Attribution License (https://creativecommons.org/licenses/by/4.0/).

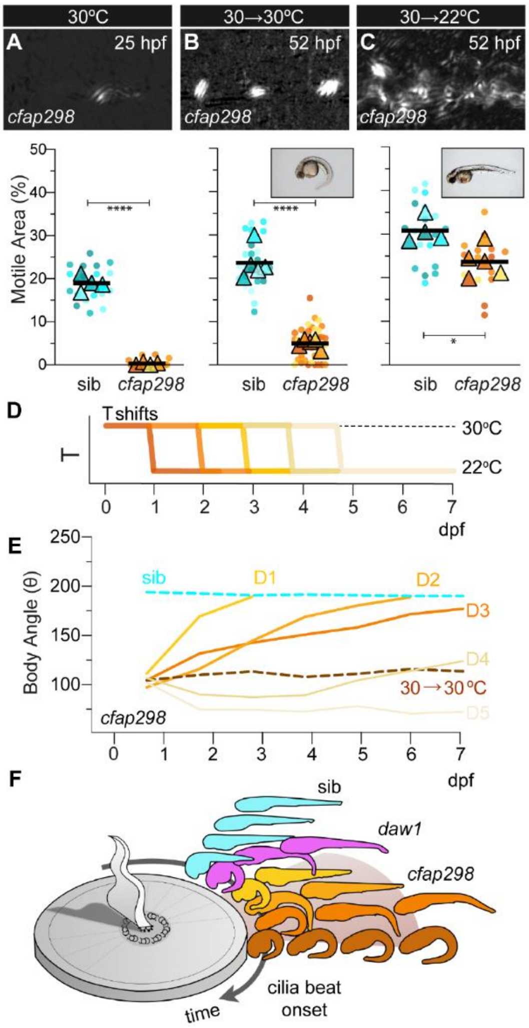

Reviewer 2 Advance Summary and Potential Significance to Field:
The manuscript by Bearce et al. document the ciliary phenotypes of daw1 mutant zebrafish. The principal discovery from their analysis is that axial curvature of zebrafish embryos can be rescued by restoration of cilia motility at later stages of development. Although the data are well documented, there is really nothing strikingly novel. Also the paper is descriptive and there are no mechanistic insights.
Furthermore, with Daw1 mutant Chlamydomonas and mice already published, the zebrafish findings presented here add marginal advance to what we already know about Daw1 function.

Reviewer 2 Comments for the Author:
My view is that this paper is not suitable for publication in Development as it does not provide any significant conceptual advancement.

We are sorry this reviewer felt our paper provided no advancement. Our finding that *daw1* mutants exhibit delay in onset of robust beating in the central canal is novel and advances what we know about Daw1. As the reviewer rightly points out, Daw1 has previously been studied in *Chlamydomonas* and mouse, but our finding of the time/stage-dependent aspect of the phenotype was not previously known.

We note that Reviewer 3 points out that that our work is novel in tackling the temporal aspect of Daw1 function.

Beyond advancing what we know about Daw1 function (which is not the only aspect of this manuscript), we used Daw1 and Cfap298 mutants to demonstrate that zebrafish can undergo a profound remodeling of their shape. How organisms can "self-correct" aberrations as they grow is an understudied aspect of morphogenesis, and our manuscript provides a clear example and, given

© 2022. Published by The Company of Biologists under the terms of the Creative Commons Attribution License (https://creativecommons.org/licenses/by/4.0/).

the many experimental attributes of the system, also a new context in which future mechanistic studies can be performed to understand how zebrafish are able to self-correct in this way. We hope this will be valuable to others working on concepts of shape remodeling and target morphology. We note that Reviewer 1 was especially excited by this aspect of our manuscript.

Reviewer 3 Advance Summary and Potential Significance to Field:
Ciliogenesis (the process of building cilia) often involves common molecular players and processes to assemble the diverse types of cilia found across cell types in metazoans and conserved to the unicellular protists. No protein synthesis occurs in cilia; everything must be synthesized in the cell body and imported through macromolecular machinery of intraflagellar transport (IFT) for delivery and incorporation at the growing cilia tip. In the case of motile cilia, in addition to the microtubule-based axoneme scaffold, large and complex (~1.5 MDa) molecular motors which power ciliary beat called dynein arms must also be transported en masse into assembling cilia. Likely cells have evolved players which can expedite or streamline efficient and timely delivery of these cargoes such that full cilia motility can be achieved by the cell within the developmental window for which this functionality required whether it be for mucociliary clearance of airways or movement of CSF within the brain ventricles.
Daw1/Oda16/Wdr69 is one such factor first identified in C. reinhardtii as a factor involved in delivery of outer dynein arms within the axoneme via interaction with the IFT-B machinery, very much different to other primary ciliary kinesia (PCD) genes which disrupt stability or assembly of these motors. Beautiful work in several papers from the Mitchell lab showed that ODA16 appears to be needed only for efficient transport of outer arm dynein motor complexes into the flagellar compartment. They also showed that 10-20% outer arm dyneins manage to assemble in the absence of ODA16 along the length of the flagellar axoneme, leading them to hypothesize that ODA16 acts as a cofactor or adaptor that enhances the ability of the IFT machinery to transport outer arm dynein. There is a considerable body of published work on Daw1/Oda16/Wdr69 in other organisms as well including zebrafish morphants and mouse point mutants showing functional conservation where ODA import and cilia motility is impaired. What is novel in this manuscript by Bearce et al is how it tackles the temporal element of ODA import and how this alters cilia motility over time. Functional diversity of cilia in metazoa is of great interest to the field, as is how cilia can be 'remodelled' over time- both concepts which have long been neglected. Using zebrafish point mutants (and crispants) of daw1, the authors use beautiful whole organism as well as organelle imaging to show both physiological as well as cellular defects are differentially dependent on daw1, some of which appear to be reversible such as the body curvature defects in embryos. The authors argue that some of these gross anatomical features can 'right' themselves over time, whilst others that occur transiently like at KV for L/R patterning or positioning of otoliths work in 'restricted' developmental windows where delays cannot be tolerated. They conclude this 'temporal restriction' is why human DAW1 patients do not have PCD rather just laterality defects in an accompanying paper. (The paper was not shared with this manuscript). Overall the data is well presented, however it currently falls short in 'mechanism' without refuting possibilities for these apparently different sensitivities.

Reviewer 3 Comments for the Author:
Major concerns
1. Dynein import versus cilia motility phenotypes across developmental time: Again 10-20% penetrance of the CTD phenotype in mutants is the starting point and (extrapolate 10-20% ODAs in oda16 mutants in Chlamy). Were the 'fully penetrant' anatomical phenotypes in the first day (otoliths and heart looping) because there was no ODAs imported (delayed) thus no motility at all in these windows, or was there some motility (10-20%), but it was not enough (physiological differences in requirements for motility)? 8 somite stage of KV cilia mostly motile in controls, what about your oda16 mutant and if not motile, how late can you see if KV cilia start to move? Is it time that is the issue or sensitivity to cilia movement (force and frequency)? Alternately could there be tissue-specific compensation in some cilia types by another factor? How long lived are cilia/ciliated cells in the central canal? The 'downshift' of the cfap298 ts allele goes some way to address this but the latest timepoint 3dpf could still be developmental plasticity- taking it out to a further timepoint would go a long way to address this. What is the latest point you could downshift at to restore body curvature? Based on your Figure 2 F'-I' it looks like cilia beat frequency/ coverage- (a term which needs better explanation- frequency and waveform may be more apropos in the field, as we know these parameters change over time in motile cilia development and coverage would like only capture how wide a cilia extends to cover the central canal as opposed beat frequency

© 2022. Published by The Company of Biologists under the terms of the Creative Commons Attribution License (https://creativecommons.org/licenses/by/4.0/).

and changes in waveform to more synchronized) is still emerging in controls at your last time point in your downshift assays but does it continue to increase and/or mature so there less spread of the distributions?
• Recommendations: Live imaging of KV cilia in controls and mutants- is motility delayed (or never starts). Clearer use of cilia beat frequency and waveform, density may not capture changes.

We agree with the reviewer that context, and physiological differences in the requirements for certain levels of motility between distinct contexts, likely contributes to the Daw1 phenotypes. Our manuscript focuses on cilia in the central canal, where we could image cilia motility over several time points as well as assess anatomical outcomes (body straightening). Due to the reviewer's suggestions, we have now imaged cilia in the KV, pronephric duct and sperm flagella from *daw1* mutants.

Based on the reviewer's suggestion, we have now imaged cilia in Kupffer's vesicle (KV) in *daw1* mutants (**Figure B** - for reviewer only). The result was that cilia were entirely immotile across the entire KV in 4/5 *daw1* mutants at the 10/11-somite stage. In 1/5 mutants, a small number of cilia showed some motility. By this time point, *dand5* is already L/R asymmetric in wild-type embryos and so the outcome of cilia-driven fluid flow has already been established. We conclude that if cilia motility is delayed in KV in *daw1* mutants, it is delayed long enough that the window when it could function has passed. This is likely because KV cilia are only present and functional for a few hours, unlike cilia in the central canal. Shortly after the stage we imaged, KV regresses and so it is not feasible to follow KV cilia for an extended period. Thus, it is impossible to know whether KV cilia are simply "delayed" in their beating or would instead never achieve functional levels of beating no matter how much time they had. The other possibility is that Daw1 is more essential in KV than in the central canal for robust motility.
Both of these possibilities are mentioned in the Discussion portions of our manuscript where we highlight that cellular context is also a potential aspect of Daw1 phenotypic differences across tissues.

In addition to KV, we imaged motile cilia in the pronephric duct in *daw1* mutants (see **new Movies 6-9**). These cilia followed a similar trend to central canal cilia, with the earlier time point (25 hpf) being more impacted than the later time point (34 hpf), suggestive of a delay in *daw1* mutants.
We extended our *cfap298* temperature shift experiments (temperature shifts now go to 5 dpf and body curve analysis to 7 dpf). The result was that the later shifts occurred, the less successfully the body could straighten. For instance, when shifting at 5 dpf, the body could not straighten at all by 7 dpf, the latest time point we could image.

We now use "motile area" as a more descriptive term than "density" and, moreover, we include kymographs and frequency quantitation (see, for example, Figures 2 and S7).

2. Citations: There is lots of exciting things in this paper but he narrative of the paper needs to be much more transparent about all that was known about ODA16/DAW1 previously, shaped around what was not known rather than present findings that support the studies that came before. For example, Oda16/ Wdr69 mutants and morphants are shown not to affect cilia length and number in fish (Gao et al 2010), planaria (Lesko et al 2020), mice (Solomon et al 2017) or Chlamy (Ahmad et al 2008) but in the 6th paragraph of the results discussion section, authors focus on the IFT46 interaction (citing the Ahmad paper) and IFT46 mutations causing shortened cilia. There is no evidence that ODA16 disrupt anterograde IFT from any of these previous studies and this line of analysis seems tangential. (And a missed talking point Esben's new paper showing differences in human and Chlamy ODA16 structure where the N-terminus that interacts with IFT46 is not conserved, other adaptors for ODA import likely to exist, possible differences between species. https://www.ncbi.nlm.nih.gov/pmc/articles/PMC7255517/) It is not until the 8th paragraph that this role for ODA16/WDR69 is discussed in more detail- this all needs to be at the front of this manuscript much more than it currently is in the introduction. Similarly, when talking about allelism in paragraph 9, the fact that in all these other systems 10-20% of ODAs still exist suggest it is required but not essential for the process and that similarly daw1b1403 is a strong loss-of-function allele, I would take out the 'null condition' as it does not add more than the previous term.
•Recommendations: Textual edits and narrative overhauls.

© 2022. Published by The Company of Biologists under the terms of the Creative Commons Attribution License (https://creativecommons.org/licenses/by/4.0/).

We have edited the text and narrative in line with the reviewer's suggestions.

We changed "Since Daw1 interacts with IFT46 and mutation of IFT46 causes shortened cilia, we quantified cilia length in *daw1*^{b1403} mutants but found no defects" to simply "Immunostaining showed the expected pattern and length of cilia in mutants (Fig. S6) as expected (Gao et al., 2010)".

We now cite the Wang et al., manuscript from Esben's lab and include more information in the introduction about Daw1, mentioning species-specific differences and the idea that Daw1 is an enhancer of ODA import:

Some species-specific differences in ODA16 homolog structures also imply possible differences in the mechanics of ODA16 acPon (Wang et al., 2020). Moreover, ODA16 is not absolutely required for moPlity in some contexts; for instance, in *Chlamydomonas*, ODA16 is not essenPal for ODA import but instead enhances the efficiency of import (Ahmed and Mitchell 2005; Ahmed et al. 2008).

We removed reference to "null condition". We also generated a new *daw1* mutant line that harbors an exon deletion and multiple premature truncation codons (described in **new Supplementary Fig 9**).
Since these mutants also exhibit CTD but straighten over time, as *b1403* mutants and *daw1* crispants did, this adds further support that we are seeing the effects of a strong Daw1 loss-of-function scenario.

Differences in cilia types and cellular sensitivities to dysfunction: It seems key to your central tenet that longer lived cilia (on longer lived cells) can improve their motility over time, whilst transient cilia (or on transient cell types) are not given the opportunity to catch up their dynein quotient necessary for 'normal' motility. The Daw1 mice showed significant mucociliary clearance defects which suggest that while motility grossly is normal (i.e. ciliary beat frequency), the necessary force generated by ODAs is subpar. Importantly they do TEM to show partial loss of ODAs in these cells, explaining this phenotype. This is what is lacking in my opinion in the paper by Bearce currently- is the content of the ODAs shifting over time, slow to accumulate on the mutant axoneme and thus slow, dyskinetic movement? Is the rate the same between diverse cilia/cell types (ei KVs versus central canal cells), just how permanent a structure it is different (i.e. longer time to catch up)?
• Recommendations: This is about import of ODAs into cilia- IF staining of KV cilia versus different timepoints in central canal, or TEM sections to show content of ODAs changing over time in different cilia types.

We agree with the reviewer that cell type-specific differences may be manifesting in *daw1* mutants. This is part of the reason we decided to initially focus on central canal cilia across time points, rather than looking at many cilia types between different tissues, which answers a different, but related question.

We have now performed imaging in both the KV and kidney as described above. To summarize, our results were:

1) KV cilia were almost entirely immotile in *daw1* mutants, implying either KV cilia are not long-lived enough to have developed motility due to delayed ODA import or KV cilia are inherently different and more impacted by *daw1* mutation for reasons beyond delayed import.
2) Kidney cilia were more impacted at earlier time points than later ones, implying a "recovery" over time, similar to what we are seeing in the central canal.

Unfortunately, we do not have working antibodies that distinguish different ODAs in the zebrafish central canal so it is not possible for us to determine which ODAs might be delayed in their import. We also do not have the technical expertise to determine whether the force generated by (otherwise normal looking) cilia beating is different in *daw1* mutants.

Our manuscript is more about linking the amount of motility to developmental outcomes, rather than understanding which specific ODAs are present in distinct contexts at different times. While very interesting, we believe this to be beyond our current scope, but we thank the reviewer for the suggestion of this exciting future avenue.

© 2022. Published by The Company of Biologists under the terms of the Creative Commons Attribution License (https://creativecommons.org/licenses/by/4.0/).

We have added much more discussion about the possibility of intrinsic cell-specific differences due to differential requirements of Daw1 function beyond this "delay" affect we are seeing in the central canal and kidney.

Minor concerns
1. Improved scholarship in introduction of cilia motility- cilia motility is powered by dynein arms which come in two flavours outer (ODAs) and inner (IDAs) which power cilia beat frequency and waveform respectively, in the abstract and introduction.

In the abstract we now say "Motility is powered by dynein arm complexes" rather than "ODAs" and then, in paragraph 3 of the introduction, we distinguish the roles of ODAs and IDAs as the reviewer suggests.

2. Abstract: change ‘accelerates’ to ‘facilitates’. Accelerates would suggest making it faster than normal and those experiments were not done. Consider changing ‘which sets the time’ to ‘promotes the onset of timely cilia motility’? Again, ‘setting the time’ is usually terminology for clock or pacemaker type functions, which this is not. Last intro paragraph ‘instead speeds us the rate at which’ consider ‘instead ensures timely and robust motility is achieved’.

All suggested changes have been made.

3. Second paragraph be specific about which species you are referring to. Cilia on multiciliated cells do not turn over gradually but the cells of those epithelia do (this comes up again in the results section). You are missing the airway cell paper Oltean paper from the Brody lab on how cilia are build, motors move in over time and beat changes- https://pubmed.ncbi.nlm.nih.gov/29851510/.

We now make clear that the multiciliated cells turnover and not the individual cilia on the cells. Thank you for pointing out the missing Oltean paper, which we now mention and cite in the Introduction.

4. Results section: ‘viable and fertile’- there are no sperm defects in the mutants- no flagellar motility defects and if so, why not?

We indeed did not observe fertility issues in either homozygous male or female *daw1* mutants. By contrast, *cfap298* mutant males are sub-fertile (Jaffe et al., 2016). We don't know the reasons for these differences, but it likely reflects cell-specific requirements for different motility factors. It could also be that Daw1 is required for robust sperm flagella motility in a time-dependent fashion, as in the central canal, with this not manifesting as sub-fertility because Daw1 sperm flagella have sufficient time to develop high levels of motility before a mating. Moreover, it is not currently clear just how much flagella motility is needed for zebrafish fertilization.

Based on this comment, we decided to extract and image *daw1* mutant sperm, indeed finding no visual differences between controls and *daw1* mutants (**new Movies 10 and 11**).

5. ‘At 25 hpf, we found disruptions to otolith positioning’ should explicitly state confirming the results of the Gao paper, similar with the L/R paper.

We now emphasize this by adding the clause “as also found in Daw1 morphants (Gao et al., 2010)”

6. Daw1 mice have significant mucociliary clearance defects, as well as high incidence of hydrocephaly supports different tissue sensitivities to cilia motility (onset) but also quality.

We now emphasize this tissue-specific effects in several places, and also mention the Solomon result of mucociliary clearance defects in *Daw1* mutant mice potential being due to reduced quality of cilia beating:

© 2022. Published by The Company of Biologists under the terms of the Creative Commons Attribution License (https://creativecommons.org/licenses/by/4.0/).

Indeed, a *Daw1* mouse mutant exhibited severe L-R patterning defects but relatively minor lung cilia motility abnormalities (Solomon et al. 2017), although mucociliary clearance was still defective in these mutants, suggestive of reduced quality of cilia beating.

7. Suggestion to discuss PCD more as a spectrum of cilia motility defects, not all tissues equally affected would be useful when you discuss your human data.

We now mention that "In addition to potentially differential effects on different organ systems of altered timing of onset of beating, PCD more generally represents a spectrum of motility defects where not all tissues are equally affected. This too likely contributes to the range of defects in distinct tissues observed upon Daw1 mutation."

8. Figure 1C-E: consider annotating what the difference is from D and E on the figure more clearly. Consider maybe adding percentages?

We have now done this to make the figure more clear.

9. Figure 2F- does not show the frequency as stated in the text- this data is important. Explain what 'cilia density' is in the legend. The results section for this figure mentions small number of cilia beating slowly and erratically but having this data in the figure would be helpful.

We have now removed "density" to describe our cilia analysis metric, and instead are using "motile area" which more accurately reflects this measure. We use a Fourier transform-based analysis that detects areas with cyclical intensity fluctuations (here corresponding with ciliary movement) and displays them as as brighter gray values compared to immotile areas. Following thresholding and binarization, we ultimately measure the area occupied by bright spots within the region of interest, as a proxy for density of *beating* cilia. It is reported as a percentage of the total image area. This is now explained in the Methods and Figure legend.

10. Figure S4: Kidney is labelled in both daw1 and foxj1a(B) but C shows no kidney expression for daw1. Maybe explain the 'differentiating neurons' track given this legend is specifically about motile cilia genes.

Part C of the figure shows a gray circle for *daw1* expression in kidney. Since the size of the circle represents the number of cells expressing *daw1* above a threshold, while the intensity of purple is the level of expression, these data suggest that *daw1* is expressed in the kidney but at lower levels than other in other tissues.

11. Figure S6: how many animals were looked at per genotype in legend.

We imaged and quantified 6 siblings and 6 mutants to generate these data, with >50 cilia per field of view, with 3-4 fields of view per individual, something we have now added to the legend.

12. Figure S7. Label your time points on the figure. What does each point refer to- one cilia frequency reading per animal or independent readings from the same animal (how many animals were looked at)?

Time points have been added to the figure and information of where readings are taken from have been added to the legend.

13. Figure S9A': Even at the non-permissive temperature a sizeable promotion of cfap298 mutants seem to catchup within a day, with this huge distribution (tail) by day 4. Are the escapers such that this is a leaky allele? Are their cilia immotile? Is this seen in your other cfap allele Figure 3? This difference is never commented on and from the M&M section they seem to be the same allele?

We agree that there is a large distribution among *cfap298* mutants kept at the restrictive temperature (28˚C) that grows larger over time. While imaging, we noted that a larger proportion of cilia are motile in those that individuals spontaneously straighten, suggesting a strong link between amount of motility and straightening.

© 2022. Published by The Company of Biologists under the terms of the Creative Commons Attribution License (https://creativecommons.org/licenses/by/4.0/).

We hypothesized that the reason for these "escapers" could be due to a contribution by maternally deposited wild type mRNA, as initial experiments were done using the progeny of heterozygous crosses. Therefore, we performed crosses using homozygous females and heterozygous males (we did not use homozygous males, due to the impaired fertility), ensuring that all maternally-deposited mRNA contained the mutant allele. By following these embryos over the course of seven days as shown in **Figure S10B**, we show that there is significantly less spontaneous straightening of embryos in these clutches, which we mention in the text. Thus, one reason for spontaneous straightening is likely caused by residual wild-type *cfap298* from the mothers.

Incomplete penetrance of the mutant allele due to its highly temperature-sensitive nature (only 6 degrees Celsius separates “restrictive” and “permissive”) could also account for some of the phenotypic variability. Due to the confounding effects of raising embryos at temperatures over 28˚C over long periods of time (off-target effects such as severe edema and premature death), we are unable to address this concern directly.

14. The last sentence in the results discussion section is challenging to comment on the validity as accompanying (submitted) manuscript was not submitted to allow review. This is unusual.

This manuscript is under review at a different journal.

Textual edits

1. Introduction, 1st paragraph: add ‘motility to rescue CTD phenotypes’ and this sentence could benefit from a little edit for clarity, maybe with ‘embryonic rescued mutants going on to develop spinal curves’?

2. Throughout- idiopathic scoliosis should be lower case throughout the manuscript.

3.Remove par excellence?

4.IFT should be lower case intraflagellar transport. We have made all of these changes.

Second decision letter

MS ID#: DEVELOP/2021/200017

MS TITLE: Daw1 Regulates the Timely Onset of Cilia Motility During Development

AUTHORS: Elizabeth A Bearce, Zoe H Irons, Samuel B Craig, Colin J Kuhns, Cynthia Sabazali, Dylan R Farnsworth, Adam C Miller, and Daniel T Grimes

I have now received all the referees reports on the above manuscript, and have reached a decision. The referees' comments are appended below, or you can access them online: please go to BenchPress and click on the 'Manuscripts with Decisions' queue in the Author Area.

The overall evaluation is positive and we would like to publish a revised manuscript in Development. As you will see Reviewer 3 still has some minor outstanding concerns that you might consider addressing in the final version of the manuscript. Your paper will not require re-review, rather I will look it over myself prior to acceptance. Please attend to all of the reviewers' comments in your revised manuscript and detail them in your point-by-point response. If you do not agree with any of their criticisms or suggestions explain clearly why this is so.

© 2022. Published by The Company of Biologists under the terms of the Creative Commons Attribution License (https://creativecommons.org/licenses/by/4.0/).

Reviewer 1

Advance summary and potential significance to field

This paper provides high-quality data about the role of Daw1 in cilia motility, and crucially, shows that body axis defects self-correct over time - a really novel and important finding in this system.

Comments for the author

This paper is further improved from the original. All of my issues are fixed and I think they've done a very nice job responding to the extensive other comments. I think it should be published now.

Reviewer 2

Advance summary and potential significance to field

I do not think there is a major advancement made in this study.

Comments for the author

No outstanding concerns.

Reviewer 3

Advance summary and potential significance to field

The paper by Bearce at all shows that delays in onset of cilia motility can have different effects in different tissues (central canal and kidney cilia), where the phenotypes namely body curvature can rectify/ameliorate over time using elegant genetics and quantitative imaging.

Comments for the author

The revised work by Bearce at el. has gone some way in addressing my concerns namely an additional deletion mutant and a prolonged analysis of the cfap298 downshift experiments to look at developmental plasticity to CTD reversal.

I am still concerned that there is a second manuscript which authors refer to as unpublished…. Usually, such manuscripts would be submitted along with the manuscript being reviewed so reviewers and editors can understand overlap/distinctiveness and survey any data which this paper references. This has not been done.
Moreover data from ‘early’ cilia timepoints in KVs is not in this paper although this seems key for their model.
Indeed there is an air of ‘carving’ up data between two stories, but using the data to reference in both but not showing it and thus limits the power of this story. It is not transparent.
As such I think the authors need to limit the title to the data available to reviewers and editors which is limited to the central canal in the title and figure legends and discussion- this is not an encompassing story of different cilia types on its own.

Points to still be addressed:
1. You need to introduce the acronym CC- currently lacking.
2. Second page of intro, last paragraph- extra space after Daw1 before comma.
3. In the results section, explicitly state that daw1b1403 homozygotes the first time you refer to the mutants for clarity.
4. As discussed above, the following statement needs clarifying to cover what is in this manuscript as opposed to the whole story the authors may not. This state for example ‘inefficient ODA import in daw1b1403 mutants causes delayed onset of robust motility, explaining why motile cilia are more affected earlier in development than later, and why early motile cilia-dependent

© 2022. Published by The Company of Biologists under the terms of the Creative Commons Attribution License (https://creativecommons.org/licenses/by/4.0/).

processes are severely impacted while later ones are not.' Explicitly state what you mean by early and late cilia phenotypes in relation to what is in this paper.
5. PCD change to primary ciliary dyskesia (not capitalized).
6. Take out the reference to unpublished data and remove the human data if this is not to be in the public domain (i.e. preprint) upon publication. Like the last sentence in the discussion.
7. Figure 1B legend- Human proteins all caps.
8. Figure 2 legend title- Since this paper focuses exclusively on curvature then amend this title on body axis to reflect that this is nothis is not in relation to laterality….(LR axis patterning which is still affected in these mutants).

Second revision

Author response to reviewers' comments

Reviewer 1

Reviewer 1 Advance Summary and Potential Significance to Field...

This paper provides high-quality data about the role of Daw1 in cilia motility, and crucially, shows that body axis defects self-correct over time - a really novel and important finding in this system.

Reviewer 1 Comments for the Author...

This paper is further improved from the original. All of my issues are fixed and I think they've done a very nice job responding to the extensive other comments. I think it should be published now.

We thank the reviewer for their constructive feedback and we are pleased they now consider the paper ready for submission.

Reviewer 2

Reviewer 2 Advance Summary and Potential Significance to Field...

I do not think there is a major advancement made in this study.

Reviewer 2 Comments for the Author...

No outstanding concerns.

We are sorry and disagree, but are pleased there are no outstanding concerns.

Reviewer 3

Reviewer 3 Advance Summary and Potential Significance to Field...

The paper by Bearce at all shows that delays in onset of cilia motility can have different effects in different tissues (central canal and kidney cilia), where the phenotypes namely body curvature can rectify/ameliorate over time using elegant genetics and quantitative imaging.

Reviewer 3 Comments for the Author...

The revised work by Bearce at el. has gone some way in addressing my concerns namely an additional deletion mutant and a prolonged analysis of the cfap298 downshift experiments to look at developmental plasticity to CTD reversal.

© 2022. Published by The Company of Biologists under the terms of the Creative Commons Attribution License (https://creativecommons.org/licenses/by/4.0/).

I am still concerned that there is a second manuscript which authors refer to as unpublished…. Usually, such manuscripts would be submitted along with the manuscript being reviewed so reviewers and editors can understand overlap/distinctiveness and survey any data which this paper references. This has not been done. Moreover data from 'early' cilia timepoints in KVs is not in this paper although this seems key for their model. Indeed there is an air of 'carving' up data between two stories, but using the data to reference in both but not showing it and thus limits the power of this story. It is not transparent.

As such I think the authors need to limit the title to the data available to reviewers and editors which is limited to the central canal in the title and figure legends and discussion- this is not an encompassing story of different cilia types on its own.

We thank the reviewer for helping make our paper stronger and are pleased that they support publication.

Points to still be addressed:

1. You need to introduce the acronym CC- currently lacking.

Fixed

2. Second page of intro, last paragraph- extra space after Daw1 before comma.

We could not find this mistake but will correct at the proof stage if it is still present.

3. In the results section, explicitly state that daw1b1403 homozygotes the first time you refer to the mutants for clarity.

Fixed

4. As discussed above, the following statement needs clarifying to cover what is in this manuscript as opposed to the whole story the authors may not. This state for example 'inefficient ODA import in daw1b1403 mutants causes delayed onset of robust motility, explaining why motile cilia are more affected earlier in development than later, and why early motile cilia-dependent processes are severely impacted while later ones are not.' Explicitly state what you mean by early and late cilia phenotypes in relation to what is in this paper.

We have now added KV imaging showing motility in WT and lack of motility in *daw1* mutants. Additionally, we have tried to make this statement as clear as possible:

We suggest that inefficient ODA import in *daw1*^{b1403} mutants causes delayed onset of robust motility, explaining why motile cilia are more affected earlier in development (i.e. in KV as well as in the central canal and pronephros at earlier time points) than later. This can also explain why early motile cilia-dependent processes like L-R patterning and initial axial straightening are severely impacted while later ones are not.

5. PCD change to primary ciliary dyskesia (not capitalized).

Fixed

6. Take out the reference to unpublished data and remove the human data if this is not to be in the public domain (i.e. preprint) upon publication. Like the last sentence in the discussion.

We would prefer to leave the minor reference to human data in our paper if acceptable by *Development*. We do not have full control over this manuscript and so we cannot preprint it. However, it has already been submitted twice to another journal and should be published shortly after this manuscript.

© 2022. Published by The Company of Biologists under the terms of the Creative Commons Attribution License (https://creativecommons.org/licenses/by/4.0/).

7. Figure 1B legend- Human proteins all caps.

Fixed

Third decision letter

MS ID#: DEVELOP/2021/200017

MS TITLE: Daw1 Regulates the Timely Onset of Cilia Motility During Development

AUTHORS: Elizabeth A Bearce, Zoe H Irons, Samuel B Craig, Colin J Kuhns, Cynthia Sabazali, Dylan R Farnsworth, Adam C Miller, and Daniel T Grimes
ARTICLE TYPE: Research Report

I am happy to tell you that your manuscript has been accepted for publication in Development, pending our standard ethics checks.

© 2022. Published by The Company of Biologists under the terms of the Creative Commons Attribution License (https://creativecommons.org/licenses/by/4.0/).
